# Evaluation of Error-State Kalman Filter Method for Estimating Human Lower-Limb Kinematics during Various Walking Gaits

**DOI:** 10.3390/s22218398

**Published:** 2022-11-01

**Authors:** Michael V. Potter, Stephen M. Cain, Lauro V. Ojeda, Reed D. Gurchiek, Ryan S. McGinnis, Noel C. Perkins

**Affiliations:** 1 Department of Physics and Engineering, Francis Marion University, Florence, SC 29506, USA; 2 Department of Chemical and Biomedical Engineering, West Virginia University, Morgantown, WV 26506, USA; 3 Department of Mechanical Engineering, University of Michigan, Ann Arbor, MI 48109, USA; 4 Department of Bioengineering, Stanford University, Stanford, CA 94305, USA; 5 Department of Electrical and Biomedical Engineering, University of Vermont, Burlington, VT 05405, USA

**Keywords:** IMU, motion capture, sensor fusion, abnormal gait

## Abstract

Inertial measurement units (IMUs) offer an attractive way to study human lower-limb kinematics without traditional laboratory constraints. We present an error-state Kalman filter method to estimate 3D joint angles, joint angle ranges of motion, stride length, and step width using data from an array of seven body-worn IMUs. Importantly, this paper contributes a novel joint axis measurement correction that reduces joint angle drift errors without assumptions of strict hinge-like joint behaviors of the hip and knee. We evaluate the method compared to two optical motion capture methods on twenty human subjects performing six different types of walking gait consisting of forward walking (at three speeds), backward walking, and lateral walking (left and right). For all gaits, RMS differences in joint angle estimates generally remain below 5 degrees for all three ankle joint angles and for flexion/extension and abduction/adduction of the hips and knees when compared to estimates from reflective markers on the IMUs. Additionally, mean RMS differences in estimated stride length and step width remain below 0.13 m for all gait types, except stride length during slow walking. This study confirms the method’s potential for non-laboratory based gait analysis, motivating further evaluation with IMU-only measurements and pathological gaits.

## 1. Introduction

The study of human lower-limb kinematics is critical for understanding and improving human function in many contexts including injury prevention, elderly fall risk, rehabilitation, and athletic performance [1,2,3,4,5,6,7,8]. Importantly, many of these contexts require studying kinematics from a wide range of gait types including abnormal gaits; for example, in clinical applications where gait analysis is utilized in identifying injury risk, assessing level of gait pathology, and informing and assessing treatment plans [5,9,10]. Such research is often conducted in controlled, laboratory environments using optical motion capture systems (MOCAP) that track the positions of reflective markers attached to the skin in order to estimate underlying bone movement. However, these traditional MOCAP methods incur many disadvantages starting with relatively high cost, long setup times, and the need for skilled researchers. These disadvantages limit the populations who might participate in and benefit from biomechanical assessments, studies, and treatments. They also limit the researcher’s ability to generalize findings from laboratory-based biomechanical studies to broad populations. Additionally, research questions may require continuous monitoring of subjects (e.g., in [11,12]) which is not possible using traditional lab-based (MOCAP) methods. Methods using body-worn inertial measurement units (IMUs) to study human kinematics address many of the disadvantages of MOCAP due to their relative low cost, ease of use, and portability to environments outside the lab. While IMU-based kinematic estimation methods exist, challenges still limit their applicability across broad studies in biomechanics.

Physics-based approaches (most common) for IMU-based kinematic estimation require a two-step process. In the first step, commonly known as the sensor to segment alignment, the physical relationships between the IMU sense frames (i.e., their xyz axes) and the bone anatomical frames are determined. In particular, this involves estimating the joint center locations and anatomical axes in the IMU sense frames. In the second step, IMU data is processed to estimate the IMU orientations and, when possible, positions. In combination, these two critical steps enable estimation of the skeletal kinematics from IMU data. Recognizing that these two steps involve a different set of challenges and are each open topics of research, the present study contributes to and focuses on the second step of this process and not the first.

One well-known challenge in estimating the IMU positions and orientations from IMU data is the need to correct for integration drift errors, caused by the integration of noisy acceleration and angular velocity data [13]. Many IMU-based methods, including commercially available solutions, critically rely on magnetometer data to correct drift errors in orientation estimates (e.g., [14,15,16,17]). However, if not properly accounted for, magnetic disturbances (e.g., frequently encountered indoors) may lead to inaccurate corrections [18,19]. Other IMU-based methods that avoid reliance on magnetometer data also exist (e.g., [20,21,22,23,24]). However, most of these are “single-joint” methods, require additional assumptions (e.g., level ground, knee acting as a pure hinge), or offer validated accuracy only for a single joint or only for normal walking gait. Importantly, these limitations yield methods whose accuracies are often application dependent [25]. Thus, for any estimation method to be utilized in a wide variety of biomechanical studies, other types of movements (beyond normal walking gait) must be evaluated; see, for example [26]. Additional studies consider short dynamic movements [27,28], calibration movements [23,29], military movements [30], stair ascent/descent [17,31,32], and skiing [33]. However, in the context of walking, few (if any) studies consider gait beyond normal speed, straight-line walking. Several recent studies use machine learning (ML) to estimate human kinematics from raw IMU signals [34,35,36]. However, they lack generalizability because they also only consider normal walking/running, are evaluated using simulated (versus measured) IMU data, and/or use data from a single IMU. Additionally, ML methods require large training sets, and success with gaits similar to those in the training set does not guarantee success on other types of gait.

Motivated by the limitations summarized above, the present study contributes a new error-state Kalman filter (ErKF) method to estimate the kinematics of the lower-limbs across a wide variety of walking gaits. In [37], we presented an ErKF method for a simplified three-body constructed model of the human lower limbs and demonstrated its success in accurately estimating joint angles, stride length, and step width over a long (ten-minute) trial. This paper extends the ErKF method in [37] for a full seven-body model, representing the feet, shanks, thighs, and pelvis. Further, advancements are made to the method to handle additional uncertainties and errors due to the complexities of biological joints and tissue (e.g., increased uncertainty in the sensor to segment alignment) that manifest in a seven-body model of human subjects (as opposed to the simplified constructed model in [37]). As noted above, this study focuses on IMU kinematic estimation and not the separate step of sensor to segment alignment which is an open research challenge in its own right [38,39,40]. Thus, in this study ErKF sensor to segment alignment is determined using MOCAP, meaning the ErKF method is not yet an “IMU-only” method. However, note that an IMU-based alternative for estimating the sensor to segment alignment parameters could be used in conjunction with the presented ErKF method to yield IMU-only estimates (albeit likely with lower accuracy).

The aims of this paper are to: (1) present a new IMU-based ErKF method for evaluating human lower-limb kinematics and (2) compare kinematic estimates from this ErKF method to those obtained from MOCAP on human subjects walking with a variety of gaits, including abnormal gaits such as side stepping. To this end, the prior ErKF method [37] is extended to a full seven-body model of the human lower limbs. Additionally, a novel joint axis measurement is developed for the hip and knee to reduce orientation drift errors without assumptions of gait type or how the hip and knee behave (e.g., hinge-like behavior or not). The method is evaluated across a wide range of six walking gaits, including abnormal gaits. Joint angles, joint angle ranges of motion, stride length, and step width estimates are compared to reference MOCAP data that is processed using two different methods.

## 2. Materials and Methods

### 2.1. ErKF Method for Seven-Body Lower-Limb Model

**Figure 1:**
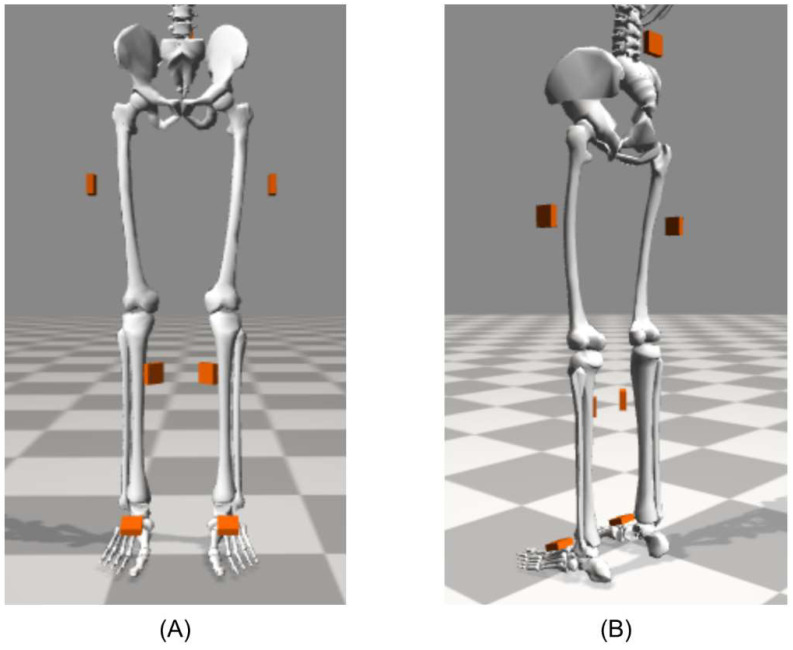
Seven-body model of the lower limbs consisting of the pelvis, thighs, shanks, and feet; visualized using OpenSim. (**A**) Front and (**B**) back views. Orange boxes represent IMU locations. Note that while OpenSim’s Gait2354 skeletal model is used here for visualization, the ErKF method treats each segment as an independent body possessing six degrees of freedom.

The underlying formulation of this ErKF method for a seven-body model of the lower limbs (refer to Figure 1) follows and extends that introduced in [37] for a simplified, three-body model of the lower limbs. The ErKF method estimates the state (position, velocity, and orientation) of each IMU (one per body) from which the lower-limb kinematics are derived via rigid body assumptions. Because the primary ErKF equations used in this study are largely the same as those in [37] (which extend from Sola’s ErKF formulation for a single IMU [41]), Section 2.1.1, Section 2.1.2 and Section 2.1.3 closely follow [37] and are included here for the reader’s convenience. In Section 2.1.4, we summarize the main differences and extensions between the method used for the 3-body lower-limb model of a mechanical walker in [37] and that for the 7-body lower-limb model of human subjects used here.

#### 2.1.1. ErKF States

Each IMU within the lower-limb model is treated as an independent (i.e., six degree of freedom) rigid body. Thus, the state for the *j*th IMU, $x_{j}$, is the (10 × 1) vector

(1) $$x_{j}=\left[\begin{array}{c} p_{j}\\ v_{j}\\ q_{j} \end{array}\right]$$

where $p_{j}$ is the (3 × 1) position vector of the IMU in a world (i.e., lab-fixed) frame, $v_{j}$ is the (3 × 1) velocity vector of the IMU, and $q_{j}$ is the (4 × 1) quaternion rotation vector (Hamiltonian convention) that relates a vector in the IMU sense frame, $y^{b}$, to its corresponding representation in the world frame, $y^{w}$, according to

(2) $$\left[\begin{array}{c} 0\\ y^{w} \end{array}\right]=q⊗\left[\begin{array}{c} 0\\ y^{b} \end{array}\right]⊗q^{*}$$

where ⊗ denotes quaternion multiplication and $q^{*}$ denotes the quaternion inverse.

In an ErKF, the filter equations are formulated with respect to the error state (i.e., errors in the state estimates), thus tracking the error state’s mean, δx, and covariance matrix, P. These error state estimates are then used to update the state estimates. The ErKF has several advantageous properties over other Kalman filter formulations for this type of application (see [41,42]). The error state for the *j*th IMU, $\delta x_{j}$, is the (9 × 1) vector

(3) $$\delta x_{j}=\left[\begin{array}{c} \delta p_{j}\\ \delta v_{j}\\ \delta \theta_{j} \end{array}\right]$$

where $\delta p_{j}$ and $\delta v_{j}$ denote errors in the position and velocity, respectively, and $\delta \theta_{j}$ is the (three-component) attitude error vector (assumed to be small) defined such that the quaternion error, $\delta q_{j}$, obeys

(4) $$\delta q_{j}=\left[\begin{array}{c} \cos\left(\frac{\left\|\delta \theta_{j}\right\|}{2}\right)\\ b\;\sin\left(\frac{\left\|\delta \theta_{j}\right\|}{2}\right) \end{array}\right]\approx \left[\begin{array}{c} 1\\ \frac{\delta \theta_{j}}{2} \end{array}\right]$$

where b is the unit vector in the direction of $\delta \theta_{j}$ (i.e., the axis of rotation) and ‖·‖ is the Euclidean vector magnitude. The full state, x, and full error state, δx, are the concatenations of those for all *n* IMUs in the system, namely

(5) $$x=\left[\begin{array}{c} x_{1}\\ x_{2}\\ \begin{array}{c} \vdots \\ x_{n} \end{array} \end{array}\right]$$

and

(6) $$\delta x=\left[\begin{array}{c} \delta x_{1}\\ \delta x_{2}\\ \begin{array}{c} \vdots \\ \delta x_{n} \end{array} \end{array}\right]$$

#### 2.1.2. Process Model

The prediction step of the ErKF uses the process model

(7) $${\hat{x}}_{j,k+1}=f\left(x_{j,k},u_{j,k}\right)$$

for each IMU where ${\hat{x}}_{j}$ denotes the prediction of $x_{j}$, the additional subscript *k* denotes the *k*th time-step, and $u_{j}$ denotes the raw IMU data (acceleration and angular velocity). Because each IMU is treated as an independent rigid body, the predicted state of each IMU at time-step k+1 is only a function of the estimated state and the IMU data at the previous time-step k. Thus, strapdown integration is used to write this process model as

(8) $${\hat{x}}_{j,k+1}=\left[\begin{array}{c} p_{j,k}+v_{j,k}\Delta t+1/2\left(R_{j,k}a_{j,k}+g\right)\Delta t^{2}\\ v_{j,k}+\left(R_{j,k}a_{j,k}+g\right)\Delta t\\ q_{j,k}⊗\left[\begin{array}{c} \cos\left(\|\omega_{j,k}\|\frac{\Delta t}{2}\right)\\ \frac{\omega_{j,k}}{\omega_{j,k}}\sin\left(\|\omega_{j,k}\|\frac{\Delta t}{2}\right) \end{array}\right] \end{array}\right]$$

where Δt is the sampling period of the IMU, R is the rotation matrix corresponding to q, g is the gravitational acceleration vector (in the world frame), $a_{j}$ is the acceleration measured by the *j*th IMU, and $\omega_{j}$ is the angular rate measured by the *j*th IMU.

As shown in [41], the mean of the error state, δx, will always be zero during the Process model in this formulation and thus does not need to be calculated. However, the error-state covariance matrix, P, is not zero and must be predicted during this step. To accomplish this, the Jacobian of the process model for the *j*th IMU at time-step *k* with respect to its error state vector, $F_{xj,k}$, is calculated as

(9) $$F_{xj,k}=\left[\begin{array}{ccc} I_{3\times 3} & \Delta tI_{3\times 3} & 0_{3\times 3}\\ 0_{3\times 3} & I_{3\times 3} & -R_{j,k}{\left[a_{j,k}\right]}_{x}\Delta t\\ 0_{3\times 3} & 0_{3\times 3} & {\left(S\left\{\omega_{j,k}\Delta t\right\}\right)}^{T} \end{array}\right]$$

where $I_{m\times m}$ represents an *m × m* identity matrix, $0_{m\times m}$ represents an *m × m* matrix of zeros, the superscript *T* denotes the transpose of a matrix, ${\left[y\right]}_{x}$ corresponds to the skew-symmetric form of y, specifically

(10) $${\left[\begin{array}{c} y_{1}\\ y_{2}\\ y_{3} \end{array}\right]}_{x}=\left[\begin{array}{ccc} 0 & -y_{3} & y_{2}\\ y_{3} & 0 & -y_{1}\\ -y_{2} & y_{1} & 0 \end{array}\right]$$

and S{w} applies the Rodrigues’ rotation formula on the vector w. To compute S{w}, w is separated into its magnitude, φ, and unit direction vector, s, to yield

(11) $$S\left\{w\right\}=S\left\{\varphi s\right\}=I_{3\times 3}\cos\left(\varphi \right)+\sin\left(\varphi \right){\left[s\right]}_{x}+ss^{T}\left(1-\cos\left(\varphi \right)\right)$$

The process noise covariance for the *j*th IMU, $Q_{j}$, is

(12) $$Q_{j}=\left[\begin{array}{ccc} 0_{3\times 3} & 0_{3\times 3} & 0_{3\times 3}\\ 0_{3\times 3} & \sigma_{a}^{2}\Delta t^{2}I_{3\times 3} & 0_{3\times 3}\\ 0_{3\times 3} & 0_{3\times 3} & \sigma_{\omega }^{2}\Delta t^{2}I_{3\times 3} \end{array}\right]$$

where $\sigma_{a}^{2}$ and $\sigma_{\omega }^{2}$ are the noise variances for the acceleration and angular rate signals, respectively.

Because each IMU’s process model is independent from that of all other IMUs, the corresponding Jacobian and process noise covariance matrix for the full system (i.e., all seven IMUs) are formed by the block diagonal matrix composition of those for the individual IMUs. Specifically, the Jacobian of the full system process model relative to the full error state at time-step *k*, $F_{x,k}$, is

(13) $$F_{x,k}=blkdiag\left(F_{x1,k},F_{x2,k},\ldots ,F_{xn,k}\right)$$

where blkdiag denotes the block diagonal matrix composition. Similarly, the full system process noise covariance matrix, Q, is

(14) $$Q=blkdiag\left(Q_{1},Q_{2},\ldots ,Q_{n}\right)$$

The prediction of the full error-state covariance matrix, $\hat{P}$, is then calculated as

(15) $${\hat{P}}_{k+1}=F_{x,k}P_{k}F_{x,k}^{T}+Q.$$

#### 2.1.3. Measurement Model

Within this ErKF formulation, known kinematic states (i.e., when the IMU is still) and constraints (i.e., relationships between the IMUs) are applied probabilistically through the measurement model to correct estimation errors. The four specific measurements used in this ErKF are described in detail below. When multiple measurements are applied during a single time step, batch processing is used to apply the measurement corrections simultaneously. Each measurement model takes the general form

(16) z=h(x)+c

where z is the observed measurement, h(x) is the expected measurement represented as a function of the state x, and c is Gaussian white noise with covariance C. Specific measurement model equations are linearized by defining the Jacobian H evaluated at x according to

(17) $$H={\frac{\partial h}{\partial \delta x}|}_{x}.$$

Consistent with [41], the chain rule decomposes H as

(18) $$H={\frac{\partial h}{\partial x}|}_{x}{\frac{\partial x}{\partial \delta x}|}_{x}=H_{x}X_{\delta x}$$

where $H_{x}$ is dependent on the specific measurement model and $X_{\delta x}$ depends only on the estimated orientation at that time. The Kalman gain, K, and error-state mean, $\hat{\delta x}$, are then calculated as

(19) $$K_{k}={\hat{P}}_{k+1}H_{k}^{T}{\left(H_{k}{\hat{P}}_{k+1}H_{k}^{T}+C_{k}\right)}^{-1}$$

and

(20) $${\hat{\delta x}}_{k+1}=K_{k}\left(z_{k}-h\left({\hat{x}}_{k+1}\right)\right).$$

The error-state mean associated with the *j*th IMU, ${\hat{\delta x}}_{j}$, updates the state mean for the *j*th IMU per

(21) $$x_{j,k+1}=\left[\begin{array}{c} {\hat{p}}_{j,k+1}+{\hat{\delta p}}_{j,k+1}\\ {\hat{v}}_{j,k+1}+{\hat{\delta v}}_{j,k+1}\\ {\hat{q}}_{j,k+1}⊗\left[\begin{array}{c} \cos\left(\|{\hat{\delta \theta }}_{j,k+1}\|/2\right)\\ \frac{{\hat{\delta \theta }}_{j,k+1}}{\left\|{\hat{\delta \theta }}_{j,k+1}\right\|}\sin\left(\|{\hat{\delta \theta }}_{j,k+1}\|/2\right) \end{array}\right] \end{array}\right]$$

where

(22) $${\hat{\delta x}}_{j}=\left[\begin{array}{c} {\hat{\delta p}}_{j}\\ {\hat{\delta v}}_{j}\\ {\hat{\delta \theta }}_{j} \end{array}\right].$$

After the state mean is updated for all IMUs, the full error-state mean is reset to zero. The error-state covariance is updated to account for the measurement(s) and the error-state mean reset per

(23) $$P_{k+1}=G_{k}\left(I_{9n\times 9n}-KH\right){\hat{P}}_{k+1}G_{k}^{T}$$

where $G_{k}$ is the Jacobian of the error-state reset operation with respect to the error state at time-step *k*, defined as

(24) $$G_{k}=blkdiag\left(G_{1,k},G_{2,k},\ldots ,G_{n,k}\right)$$

where

(25) $$G_{j,k}=\left[\begin{array}{ccc} I_{3\times 3} & 0_{3\times 3} & 0_{3\times 3}\\ 0_{3\times 3} & I_{3\times 3} & 0_{3\times 3}\\ 0_{3\times 3} & 0_{3\times 3} & I_{3\times 3}-{\left[\frac{1}{2}{\hat{\delta \theta }}_{j,k+1}\right]}_{x} \end{array}\right].$$

Note that the process and measurement models are applied for each time step. If no measurements are observed during a time step, the equations

(26) $$x_{k+1}={\hat{x}}_{k+1}$$

(27) $$P_{k+1}={\hat{P}}_{k+1}.$$

are used in place of the measurement model equations.

The four specific measurement corrections used in the ErKF consist of zero-velocity update (ZUPT), gravitational tilt, joint center, and joint axis corrections. The specific equations for these corrections are detailed next.

*Measurement Model 1: ZUPT correction.* When a foot IMU is (nearly) still (i.e., at some point during stance), a zero-velocity update (ZUPT) measurement is utilized to correct error in the estimated velocity. Many studies demonstrate that such ZUPT corrections yield accurate foot trajectory estimates across a wide variety of gait speeds [26,43,44]. For the present ErKF formulation, the measurement equation is written as

(28) $$h_{ZUPT}\left(x\right)=v_{IMU}$$

where $h_{ZUPT}\left(x\right)$ is the expected measurement of foot velocity and $v_{IMU}$ is the (3 × 1) velocity vector for a foot-mounted IMU. This measurement is applied when the foot IMU is determined to be momentarily still, yielding the (virtual) observed measurement of foot velocity

(29) $$z_{ZUPT}=\left[\begin{array}{c} 0\\ 0\\ 0 \end{array}\right].$$

*Measurement Model 2: Gravitational tilt correction.* When any IMU is determined to be momentarily still, its accelerometer gives an estimate of the direction of the gravitational vector. Thus, a gravitational (tilt) correction can be applied for the IMU orientation. Assuming an xyz world-frame convention where gravity acts in the −z direction, this correction yields the measurement model

(30) $$h_{tilt}\left(x\right)=R^{T}\left[\begin{array}{c} 0\\ 0\\ 1 \end{array}\right]$$

where $h_{tilt}\left(x\right)$ is the expected measurement of the gravitational direction (unit vector) in the IMU frame and R is the rotation matrix representing the orientation of the still IMU. Note that Equation (30) can easily be modified when using other world-frame conventions. The observed tilt measurement is

(31) $$z_{tilt}=\frac{a}{\left\|a\right\|}$$

where a is the IMU-measured acceleration.

*Measurement Model 3: Joint center correction.* At all times, it is assumed that a world-resolved joint center location must be the same as estimated by the two segment IMUs adjacent to that joint [45]. Thus, for IMUs on adjacent limbs 1 and 2, the measurement equation takes the form

(32) $$h_{JC}\left(x\right)=\left(p_{1}+R_{1}r_{1}\right)-\left(p_{2}+R_{2}r_{2}\right)$$

where $h_{JC}\left(x\right)$ is the expected difference between the joint center locations, the subscript i=1,2 denotes *IMU_i_*, $r_{i}$ denotes the known position of the joint center from *IMU_i_* (in the IMU frame), and $R_{i}$ denotes the rotation matrix (i.e., orientation) for *IMU_i_*. The (virtual) observed measurement for the difference between the joint center locations is

(33) $$z_{JC}=\left[\begin{array}{c} 0\\ 0\\ 0 \end{array}\right].$$

*Measurement Model 4: Joint axis correction.* At times, a joint is expected to have certain axes of the adjacent limbs approximately aligned in the world frame (e.g., the flexion/extension axes of the shank and thigh when the knee acts as a hinge [20,21]). This leads to the measurement model

(34) $$h_{JA}\left(x\right)=R_{1}e_{1}-R_{2}e_{2}$$

where $h_{JA}\left(x\right)$ is the expected difference between the joint axis vectors, the subscript i indexes the two adjacent limbs, and $e_{i}$ is the aligned joint axis (unit vector) for limb i in the frame of *IMU_i_*. The (virtual) observed measurement for the difference between the joint axis vectors is

(35) $$z_{JA}=\left[\begin{array}{c} 0\\ 0\\ 0 \end{array}\right].$$

#### 2.1.4. Details of ErKF Method Specific to Human Subjects

In the present method, zero-velocity update (ZUPT) measurements are applied to the foot-mounted IMUs at detected footfalls, gravitational tilt correction measurements are applied to any IMU that is detected to be nearly still, and joint center correction measurements are applied at all time steps for all (six) joints. Note that in this study we refer to near-still instances of the foot where a ZUPT correction is applied as “footfalls” (one per stance phase) to avoid confusion with other still instances (i.e., where gravitational tilt corrections are applied). Measurement noises are summarized in Table 1 and are generally the same as those used in [37], except the process model noises are adjusted to reflect the sampling rate used in the present study (128 Hz) and the hip joint axis correction noise reflects the “soft” hinge constraint described below.

In contrast to the three-body mechanical model in [37], the joints of the human lower limbs are not single degree of freedom joints, necessitating a modified approach for the joint axis correction measurements for the seven-body model. Similar to [20,21], our method exploits the fact that the knee predominantly acts like a hinge (i.e., small internal/external rotation and abduction/adduction) during normal gait. Unlike [21], where a hinge constraint is applied only during periods of detectable hinge-like knee movements, the present method assumes a “soft” hinge constraint at all time steps, assuming the knee flexion/extension axes, as estimated separately from the thigh and shank, remain generally aligned. This correction respects the fact that the knee predominantly acts in flexion/extension while still enabling measurements in the other two rotational degrees of freedom. The mathematical formulation for this measurement correction is the same as detailed above (Measurement Model 4). Because this constraint for human subjects is an approximation, a larger measurement noise may be used (depending on the hinge-like nature of a particular joint) than in [37] rendering this a “soft” constraint.

Unlike the three-body model of the walker [37], the seven-body model of the human considers soft-tissue deformations of the lower limbs. In particular, soft tissue of the thigh allows significant relative motion between the thigh IMU and the underlying femur. When this movement is ignored (as in the ErKF formulation used here), the hip joint center measurement induces significant bias and/or drift in the estimated hip joint angles as described next, and particularly the internal/external rotation. The joint center measurement correction relies on accurate estimates of the joint center locations in the IMU frames (obtained from the sensor to segment alignment) to accurately enforce the kinematic constraints. Additionally, we assume the sensor to segment alignment is constant; however, soft tissue motion causes the sensor to segment alignment to be time-variant which leads to inaccurate corrections and thus the aforementioned bias or drift in hip joint angle estimates. To diminish this effect, a joint axis correction measurement is employed at all times for the hip that mimics the “soft” hinge constraint for the knee. However, because the hip exhibits full three degree of freedom rotations during gait, a much higher measurement noise is used (57.3 deg for the hip versus 1.15 deg for the knee, refer to Table 1) for the hip joint axis correction compared to the knee. As with the knee, this measurement aids in constraining the estimated hip joint angles to anatomically realistic ranges while permitting three degree of freedom rotations. Note that no joint axis measurement corrections are used for the ankle because the ankle joint angle estimates are typically constrained to anatomically realistic ranges without such a correction.

### 2.2. Human Subject Experiment

**Figure 2:**
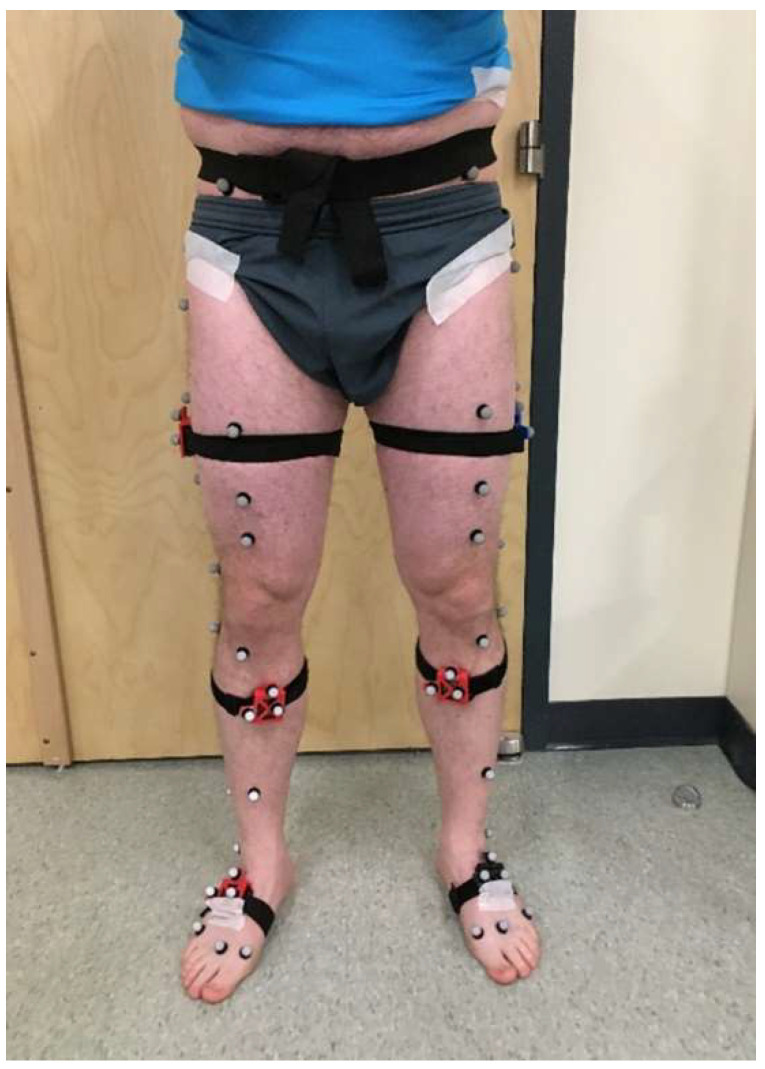
IMU and reflective marker placement for human subject experiments.

Twenty-three healthy adult subjects (inclusion: ability to perform basic tasks of daily living; exclusion: diagnosis of a balance or mobility impairment, inability to perform experimental tasks without assistance, opioid-dependence) participated in a University of Vermont Institutional Review Board approved study (protocol code #08-0518). All subjects gave written informed consent before participating in the study. Subjects wore IMUs (Opal, APDM, ±16 g and ±200 g accelerometers, ±2000 deg/s gyros) and reflective motion capture (MOCAP) markers (14 mm). Importantly, the study used a custom MOCAP marker set with markers shown in Figure 2 which includes markers placed on bony landmarks (ASIS, PSIS, lateral femoral epicondyle, fibular head, tibial tuberosity, lateral malleolus, heel, and the second metatarsal for all trials; medial femoral epicondyle, greater trochanter, medial tibial condyle, medial malleolus, and first and fifth metatarsals for calibration), markers placed on other locations shown, and three markers on each IMU, enabling two different methods for comparisons to MOCAP estimates (detailed in Section 2.6, “Estimated kinematics from two MOCAP methods”). Markers were tracked using a 19-camera system (Vero V2.2 cameras, Vicon, Oxford, UK). Subjects performed various activities of daily living in a laboratory including the six walking gaits described below. MOCAP data and IMU data were collected synchronously at 100 Hz and 128 Hz, respectively. Some data files from three subjects were either missing or created incorrectly, yielding data from twenty subjects analyzed in the present study (11 female, 9 male; mean (standard deviation) age 22.7 (±5.5), height 1.73 (±0.09 m, height not available for one subject), mass 70.3 (±12.7) kg).

Measurements from the following activities are used in this study: (1) static standing calibrations (three seconds), (2) functional calibrations (set of movements including a modified version of the StarArc hip calibration movements [29,46], knee flexions, and ankle flexions and rotations; performed on both sides), and (3) six constant-speed walking gaits on a treadmill. The treadmill walking gaits include separate trials of forward walking at three speeds (slow, normal, and fast), backward walking, lateral left walking, and lateral right walking. Each walking trial lasted one minute at a self-selected speed. Additionally, for all trials, the subject began standing on the side rails of the treadmill and transitioned to the treadmill belt within the first five seconds. Only data after both feet have left the railing are used to evaluate gait. For normal walking, only nineteen subjects are analyzed due to missing marker data for one subject. For fast and lateral left walking, only nineteen subjects are analyzed due to obvious belt speed changes during the trial for one subject each; thus, these trials are not at constant speed. For slow walking, only eighteen subjects are analyzed for the following reasons. For one subject, the belt speed obviously changed during the trial. For a second subject, the walking speed was particularly slow (<0.2 m/s) and deemed an extreme outlier.

### 2.3. Kinematic Comparisons

To evaluate the performance of the ErKF method, we compare relevant kinematic measures (e.g., joint angles, stride lengths) estimated by the ErKF method to those estimated using two MOCAP-based methods, which is considered the gold standard for clinical gait analysis. Root mean square (RMS) differences between kinematic estimates from the ErKF method and each MOCAP method are calculated for each subject and trial. We report the mean and standard deviation of these RMS differences across all subjects and separately for each type of gait. Additionally, Bland–Altman plots [47] are used to assess agreement between the ErKF method and a MOCAP method for select metrics that can be obtained from joint angle waveforms (e.g., mean knee range of motion).

In order to facilitate direct comparison of the estimation methods, a similar underlying skeletal model (i.e., same segment lengths and joint center locations) is used for both IMU-based and MOCAP-based methods. OpenSim’s Gait2354 model [48,49] is used as the base human skeletal model, but with the knee joint modified to allow three degrees of rotational freedom. Note, this model also allows three degrees of rotational freedom for the hip and two for the ankle. This skeletal model is scaled for each subject using a procedure detailed later in Section 2.4, “Calibration of ErKF and MOCAP models”. All joint angles are calculated according to the ISB recommended conventions [50,51] with the modification proposed by Dabirrahmani and Hogg [52] and based on the anatomical frame conventions defined for the Gait2354 model.

In some trials, a simple offset (bias difference) is observed between the joint angles estimated from these two modalities (ErKF and MOCAP) despite otherwise highly consistent estimates of the underlying joint angle waveforms. Consequently, we also report the range of motion of the joint angles for each stride since (1) it is highly relevant for biomechanical studies (e.g., [53,54,55]), and (2) it is also a measure of consistency of the underlying waveforms. For each joint angle, the range of motion is calculated as the difference between the maximum and minimum value of the joint angle during that stride (i.e., between successive footfalls). Range of motion is not reported for any stride should any of the associated joint angle data be missing during that stride (e.g., due to marker occlusion). Additionally, if range of motion estimates are not reported for more than 30% of the strides during a trial, no summary statistics for range of motion are reported for that trial.

**Figure 3:**
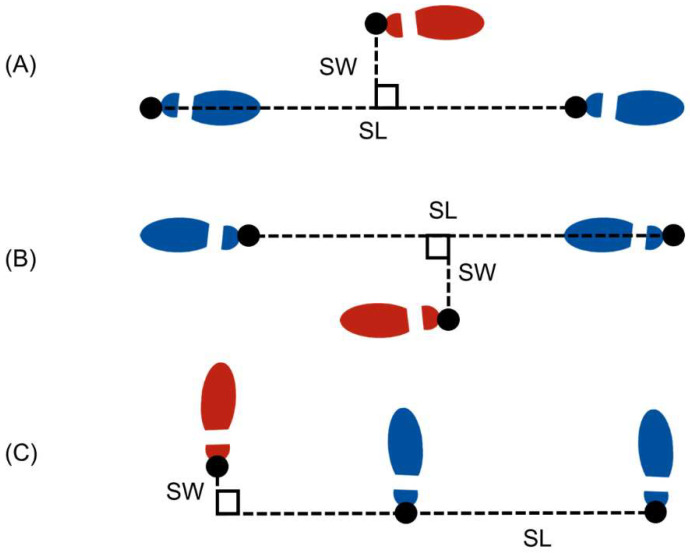
Stride length (SL) and step width (SW) definitions for forward walking (**A**), backward walking (**B**), and lateral walking (**C**). Shown in each subfigure are two consecutive right footfall locations (blue) with the intermediate left footfall location (red) for a subject traveling to the right. SL is the total horizontal displacement between consecutive footfalls of the same foot and SW is the orthogonal distance between the stride length vector and the intermediate footfall location of the opposite foot.

Similar to [37,56], stride length is calculated as the total horizontal displacement of the heel between consecutive footfalls of the same foot and step width is calculated as the orthogonal distance between the stride length vector and the heel location of the opposite footfall during the intermediate footfall. These definitions are illustrated in Figure 3 for forward, backward, and lateral walking. The first stride and last two strides represent transition strides during a trial, and they are not included in the reported stride length and step width results.

### 2.4. Calibration of ErKF and MOCAP Models

Both the IMU (ErKF) and MOCAP-based methods require determination of the mapping between the skeletal model and the IMUs and markers, respectively. MOCAP data during the static calibration and star calibration movements are used to determine both mappings as follows.

Joint centers of the hips, knees, and ankles are estimated during the static calibration as follows. Hip joint centers are calculated following Hara et al. [57] using the two ASIS and two PSIS markers to determine the pelvic frame and the average distance between the ASIS and medial malleoli to determine the leg length. The knee joint center is estimated following Davis et al. [9] as the midpoint of the lateral and medial femoral epicondyle markers. The ankle joint center is estimated following the recommendation of Siston et al. [58] as the midpoint of the lateral and medial malleoli markers.

OpenSim is then used to scale the base skeletal model to each subject using marker data from the static calibration trial, including the appended joint center estimates. OpenSim’s scale tool also determines the location of each marker in its parent segment’s frame using the same static calibration MOCAP data and the scaled skeletal model. The three markers attached to each IMU (Figure 2) define an IMU cluster frame and are assigned to the IMU’s respective parent segment. Thus, the rotation matrix from each segment’s anatomical frame to the attached IMU’s marker cluster frame, $R_{A}^{C}$, is determined from the IMU marker locations in the parent segment’s frame. To obtain the rotation matrix from the segment’s anatomical frame to the IMU sense frame, the cluster to sensor frame rotation matrix for each segment, $R_{C}^{S}$, must first be calculated. As done in [29], $R_{C}^{S}$ is computed using the procedure of Challis [59] and comparing the raw IMU angular velocity data to the estimated angular velocity of the cluster frame (i.e., calculated by differentiating the MOCAP-determined cluster orientations) using the data from the star calibration trial. The rotation matrix from the anatomical frame to the IMU sense frame, $R_{A}^{S}$, is then calculated as

(36) $$R_{A}^{S}=R_{C}^{S}R_{A}^{C}.$$

The location of each joint center in the IMU frame is determined from the static calibration trial as well as the location of heel markers in their respective foot IMU’s frame. The rotation matrices between IMU and associated anatomical frames and the joint center locations in the IMU frames make up the sensor to segment alignment required for the seven-body ErKF method.

Note that, in this study, MOCAP data are utilized for these calibrations in the ErKF method; thus, it is not yet an IMU-only method. This decision was made for the following reasons. First, because both IMU- and MOCAP-derived joint kinematic estimates are impacted by the determination of the anatomical axes and joint center locations, the IMU and MOCAP methods are more directly compared by controlling for these parameters (i.e., using similar underlying skeletal models). Second, accurately determining these calibration parameters from IMU data alone remains an open challenge in its own right [38,39,40]. Thus, using MOCAP data to establish these calibration parameters enables more direct evaluation of the ErKF method itself (i.e., independent of the input parameters) by reducing the risk of obtaining inaccurate calibration parameters which may significantly affect kinematic estimates.

In summary, the calibration procedures described above establish the required mappings between sensors/markers and the underlying bones for both ErKF and MOCAP methods. More precisely, for the ErKF method, these mappings are required inputs for the joint center and joint axis measurement corrections and critical to estimating segment poses (and thus the lower-limb kinematics) from the estimated IMU poses. In the MOCAP methods, these mappings are critical to estimating the skeletal poses from individually tracked markers.

### 2.5. Estimated Kinematics from the ErKF Method

The ErKF method yields estimates of major kinematical variables including the three-dimensional angles across all six skeletal joints as well as the stride length and step width. The method begins with estimating the positions and orientations of the IMUs (and thus the seven body segments) throughout each trial. MOCAP data are used to estimate the initial pose of each IMU (after both feet are off the rails) for establishing the initial states of the seven body segments for the ErKF. As with the model calibration step, utilizing MOCAP data for these initial poses enables more direct comparison of the IMU and MOCAP-based methods by reducing errors in the initial pose estimates. Still periods for all IMUs are determined using the same criteria as for the experimental walker in [37], but with the angular velocity magnitude threshold set to 60 deg/s. Footfall instances are identified from IMU data during each detected stance. ZUPT and tilt measurement corrections are applied at identified footfall and still period instances, respectively. After IMU poses are estimated through the ErKF, the sensor to segment alignment parameters are utilized to estimate the segment orientations from estimated IMU poses throughout the trial which are then used to estimate the three-dimensional joint angles across the hips, knees, and ankles. The joint angle estimates are then low-pass filtered using a zero-lag 4th order Butterworth filter at 6 Hz to parallel the filtering used for MOCAP estimates (described below), enabling direct comparison between the methods. To estimate stride metrics, each heel trajectory is estimated using its respective foot’s estimated IMU pose combined with knowledge of the heel marker location with respect to the IMU frame (per the above calibration procedure). Estimated heel locations at identified footfalls are then used to calculate stride lengths and step widths as previously described above in Section 2.3, “Kinematic comparisons”.

### 2.6. Estimated Kinematics from Two MOCAP Methods

The estimated kinematics from the ErKF method are compared to those estimated from MOCAP. Estimates of stride length and step width are obtained from heel marker locations at IMU-identified footfall instances. To reduce noise in the measured trajectories, heel marker trajectories in the lab frame are low-pass filtered using a zero-lag 4th order Butterworth filter at 6 Hz. Because some trajectories are missing data (i.e., due to marker occlusion), the filter is applied individually to each continuous segment of trajectory data. However, applying the filter to segments that are too short may lead to erroneous results; thus, continuous segments shorter than 0.2 s are removed. Finally, short gaps in marker trajectories (less than 0.1 s) are filled with cubic splines. Recall that the ErKF method estimates positions relative to the treadmill belt frame (due to the ZUPT measurement model), whereas the MOCAP method estimates positions in the lab frame. To compare the results from these two measurement modalities, the MOCAP-based trajectories are converted to the treadmill frame as follows. Because independent belt speed measurements are not available for all trials, the average velocity of the foot IMU markers during the first two stance phases is used as the estimated belt speed, which is assumed to remain constant. The distance the belt has traveled at each instant is then estimated by multiplying the estimated belt speed by time and that distance is added to the heel position (in the direction of travel) to estimate the heel trajectory in the belt frame. MOCAP estimates of stride length and step width are then estimated using the heel marker positions in the belt frame at the IMU-identified footfall instances.

**Figure 4:**
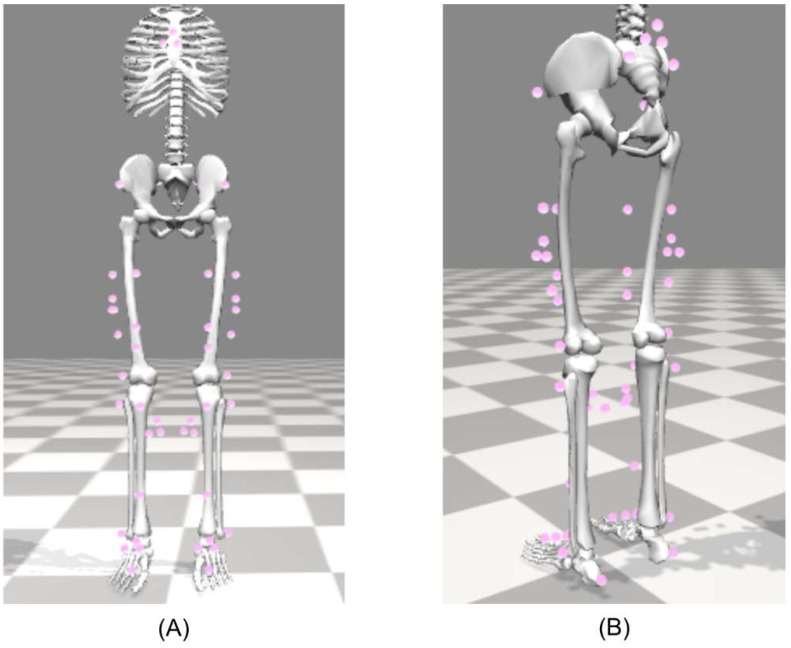
Marker locations (pink dots) used for MOCAP comparisons, visualized using OpenSim. (**A**) Front and (**B**) back views.

Two different MOCAP-based methods are employed for estimating the joint angles during walking, using the markers shown in Figure 4. For both MOCAP-based methods, all IMU marker trajectories are low-pass filtered using a zero-lag 4th order Butterworth filter at 6 Hz. Due to marker occlusion, segments of missing marker data are removed and repaired as described above for the heel trajectories. Individual details of these two MOCAP-based methods follow.

#### 2.6.1. Cluster Method (Clust)

The first method, called the cluster method, provides estimates that more closely capture the motion of each IMU because it employs the marker data solely from the IMU-mounted markers (i.e., the marker clusters). In this method, the joint angles are estimated based solely on the estimated orientations of the IMU marker clusters. This method is expected to yield estimates nearer to the ErKF method because any soft tissue motion affects the motion of both the IMU and attached cluster markers equally. However, we do not expect them to be identical because this soft tissue motion does not affect each method equally (i.e., violations of rigid segment assumptions affect estimates differently in the two methods).

Whenever all three markers on an IMU are observable, their positions determine a cluster-based orientation of the IMU frame from which the corresponding body segment’s orientation is estimated via the previously computed segment to cluster rotation matrix. Joint angles are then calculated from the estimated segment orientations. Note that if marker positional data of any of the six IMU markers adjacent to a joint are missing, no angles across the intervening joint can be calculated at that time step.

#### 2.6.2. Inverse Kinematics Method (IK)

The second method, called the inverse kinematics method, utilizes all marker locations shown in Figure 4 (i.e., both bony landmark and IMU markers) along with the scaled skeletal model (refer to Section 2.4, “Calibration of ErKF and MOCAP models”) with its associated kinematic constraints to solve an inverse kinematics problem that estimates the lower-limb kinematics [49]. The inverse kinematics tool within OpenSim is utilized to solve for all segment orientations using all observed markers and the subject-specific skeletal model. To ensure good inverse kinematics solutions, marker weightings are chosen such that marker errors generally remain below 1 cm RMS error and 4 cm maximum lower-limb marker error for each trial per recommendations in the OpenSim documentation [60]. Next, the joint angles are calculated from these segment orientations. Finally, the joint angle estimates are low-passed using a zero-lag 4th order Butterworth filter at 6 Hz.

## 3. Results

### 3.1. Results across All Twenty Human Subjects

We first report the overall performance of the ErKF method across all twenty subjects and across all joints (hips, knees, and ankles) and all six types of gait before more closely examining representative results on a single subject in Section 3.2, “Representative results on a single subject” (refer to the Supplementary File S1 for results for each individual subject). To begin, we focus on the performance of the ErKF method compared to the cluster method for the joint angles. The RMS difference between the ErKF and cluster estimate of each joint angle (flexion/extension, FE; internal/external rotation, IE; abduction/adduction, AbAd; dorsiflexion/plantarflexion, DP; inversion/eversion, InEv; positive/negative reported values) is calculated for each subject and trial. Table 2 reports the mean and standard deviation of the RMS differences across all subjects and separately for each type of gait. The green, yellow, and red highlighting denotes mean RMS differences less than 5 deg, less than 10 deg, and greater than 10 deg, respectively. Note that mean RMS differences are generally less than 5 degrees for FE (DP for ankle) and AbAd (InEv for ankle) across all joints and across all types of gait. By contrast, mean RMS differences are typically higher for IE joint angles across all gait types, except for the ankle.

Next, we evaluate the performance of the ErKF method compared to the inverse kinematics method for joint angle estimates. Table 3 reports the RMS differences between the ErKF and inverse kinematics estimates of each joint angle across all subjects and separately for each type of gait. Note that mean RMS differences generally remain less than 5 degrees for AbAd (InEv for ankle) across all joints and across all types of gait. By contrast, mean RMS differences are typically higher for FE (DP for ankle) and IE joint angles across all gait types, except FE for the knee and IE for the ankle.

Table 4 compares the range of motion estimates from the IMU ErKF method and MOCAP cluster method. This table reports the mean (and standard deviation) of the (stride by stride) RMS differences in range of motion for each joint angle across all subjects and separately for each type of gait. In the vast majority of trials, frequent marker occlusion (especially of shank IMU cluster markers) precluded estimates of knee and ankle range of motion using the cluster method. Thus, we only report range of motion differences for the hip joint angles here. While such occlusion also affected hip range of motion estimates using the cluster method, these estimates were successfully obtained for at least ten subjects for each hip and gait type (i.e., minimum of ten subjects represented in each entry of Table 4).

Next, we compare the range of motion estimates of the ErKF method and the inverse kinematics method in Table 5. This table reports the mean (and standard deviation) of the (stride by stride) RMS differences in range of motion for each joint angle across all subjects and separately for each type of gait. Unlike the cluster method, the inverse kinematics method is capable of estimating joint kinematics even when some markers are occluded. Thus, range of motion is successfully estimated for all joint angles in each trial as reflected in Table 5.

We also compare key stride metrics, namely stride length (SL) and step width (SW), estimated by the ErKF method to those estimated using the MOCAP heel trajectories. Table 6 reports both the mean (and standard deviation) SL and SW from MOCAP as well as the mean (and standard deviation) of the RMS differences in SL and SW between the two methods across all subjects and separately for each gait type. Mean RMS differences in stride length are 0.07 and 0.05 m (6% and 3.4% of the mean), respectively for normal and fast walking. Mean RMS differences in stride length for both lateral walks also remain below 0.08 m (below 16% of the mean), noting the mean stride length is much smaller than for the forward walks. For normal and fast walking, mean RMS differences in step width are 0.05 m (~45% of the mean). Note that for both stride length and step width, mean RMS differences for forward and backward walking are much smaller for the faster gait speeds (>0.8 m/s) compared to the slower speeds (<0.5 m/s).

**Figure 5:**
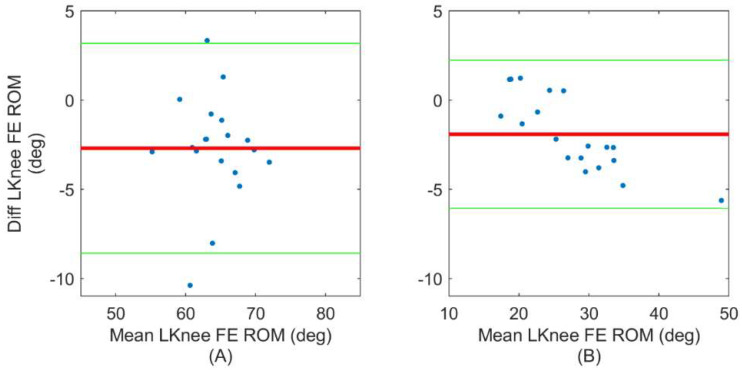
Bland–Altman plots of the left knee mean flexion/extension (FE) range of motion during (**A**) normal walking and (**B**) lateral left walking. Comparison is between IMU and IK methods (IMU-IK). Plot represents matched estimates for all 19 subjects evaluated in these movements. Bias (thick, red) and 95% limits of agreement (thin, green) are also displayed. Limits of agreement calculated as Bias ± 1.96 SD where Bias is the mean and SD is the standard deviation of the differences in the estimates.

**Figure 6:**
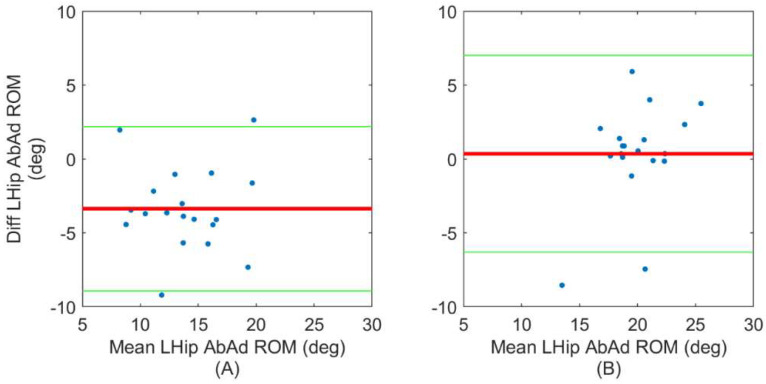
Bland–Altman plots of the left hip mean abduction/adduction (AbAd) range of motion during (**A**) normal walking and (**B**) lateral left walking. Comparison is between IMU and IK methods (IMU-IK). Plot represents matched estimates for all 19 subjects evaluated in these movements. Bias (thick, red) and 95% limits of agreement (thin, green) are also displayed. Limits of agreement calculated as Bias ± 1.96 SD where Bias is the mean and SD is the standard deviation of the differences in the estimates.

Finally, Bland–Altman plots are used to evaluate the agreement between ErKF and IK estimates of two exemplary metrics that are derived from the joint angle waveforms. First, we present Bland–Altman plots for estimates of each subject’s mean left knee FE range of motion in Figure 5 for normal (Figure 5A) and lateral left walking (Figure 5B). This metric is selected because FE range of motion is relevant in many clinical settings [61,62]. Both Figure 5A,B demonstrate good agreement between the ErKF and IK methods for estimating this metric despite the very different gait type represented in each. We also present Bland–Altman plots for estimates of each subject’s mean left hip AbAd range of motion in Figure 6 for normal (Figure 6A) and lateral left walking (Figure 6B). This case represents an extreme condition because: (1) lateral left walking induces significantly more hip AbAd than normal walking and (2) the hip clearly does not act as a hinge during lateral left walking, but the ErKF method applies a “soft” hinge-like correction at all times. Even with these considerations, Figure 6 demonstrates close agreement of the left hip AbAd range of motion estimates in both movement conditions.

### 3.2. Representative Results on a Single Subject

**Figure 7:**
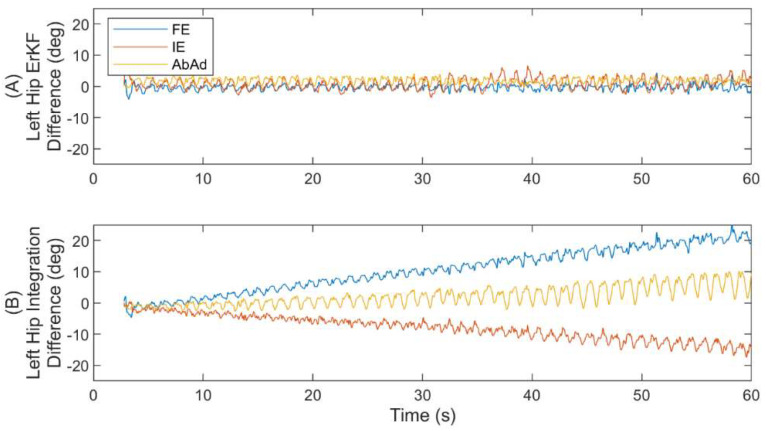
Representative left hip joint angle differences (IMU-Clust) versus time as estimated by ErKF (errors models employed) and raw integration (no error models employed). (**A**) the ErKF method and (**B**) raw integration (no filtering). The three joint angles include hip flexion/extension (FE), internal/external rotation (IE), and abduction/adduction (AbAd).

We next examine representative results on a single subject. These sample results are selected to highlight key points about the performance of the ErKF method in estimating joint kinematics. We start by comparing the differences (compared to the cluster method) in joint angle estimates for the left hip using raw integration of IMU data (no error models employed) versus application of the ErKF method (with all error models employed). We note that it is often recommended that a static period be utilized to correct for static bias in the angular rate signals to reduce the rate of orientation drift due to raw integration [63]; however, this strategy could not be employed in the present method because the validation data set does not include sufficiently long still periods to estimate the gyroscope bias. Despite this, the results in Figure 7 demonstrate no observable drift for the ErKF method in any of the three estimated joint angles for the left hip over the one-minute trial while raw integration (i.e., without the ErKF) results in differences greater than 20 deg over this same time due to drift.

**Figure 8:**
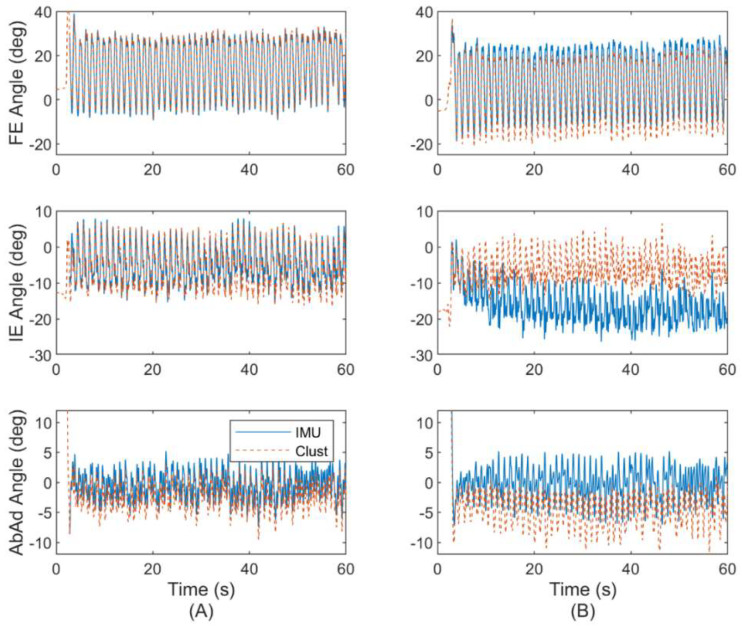
Comparison of estimated hip joint angles for representative subject between hips. Estimated hip joint angles for the left (**A**) and right (**B**) hips. Comparison is between ErKF (IMU) and cluster (Clust) methods. Offsets between estimation methods may converge despite error sources (**B**).

We next report in Figure 8 the estimated hip joint angles based on the ErKF (IMU) and cluster (MOCAP) methods for the same subject and trial considered previously in Figure 7, but now for both the left (Figure 8A) and right (Figure 8B) hips. Note that different sized offsets may occur depending on the hip (i.e., right versus left) and joint angle, but these offsets typically converge.

**Figure 9:**
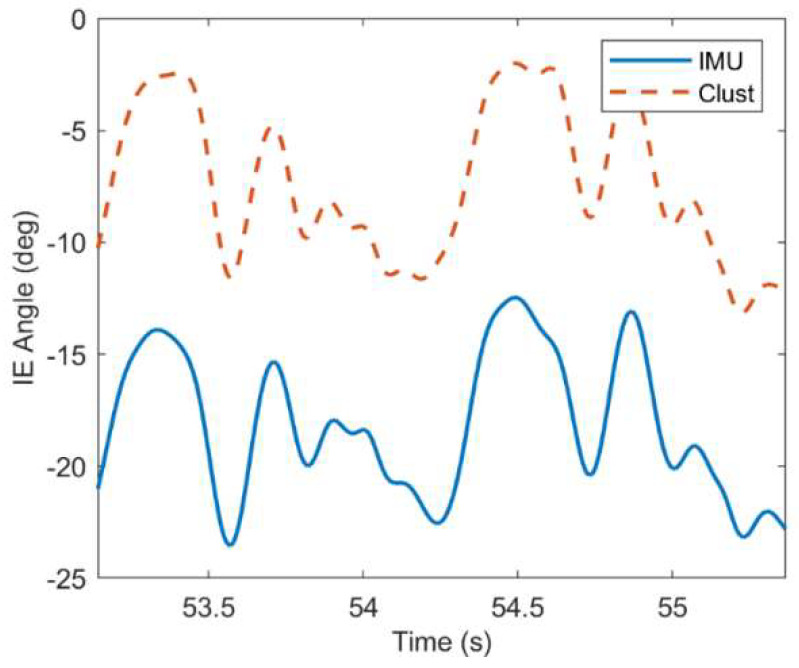
Magnified comparison of estimated right hip IE angle for representative subject in normal walking over two gait cycles. Comparison is between ErKF (IMU) and cluster (Clust) methods. Joint angle waveform characteristics, including stride to stride range of motion, estimated similarly despite offset.

Such offsets are commonly observed in this study, but vary in size depending on the subject and joint angle. Typically, the offsets (when they do arise) are smaller for FE and AbAd angles (<10 deg) than for IE angles (<30 deg) as shown in Figure 8B. Despite such offsets, the joint angle waveforms are often similar between ErKF and MOCAP methods. For example, Figure 9 shows a similar waveform between the ErKF and cluster estimates of the right hip IE angle over two stride cycles (zoom in from Figure 8B).

**Figure 10:**
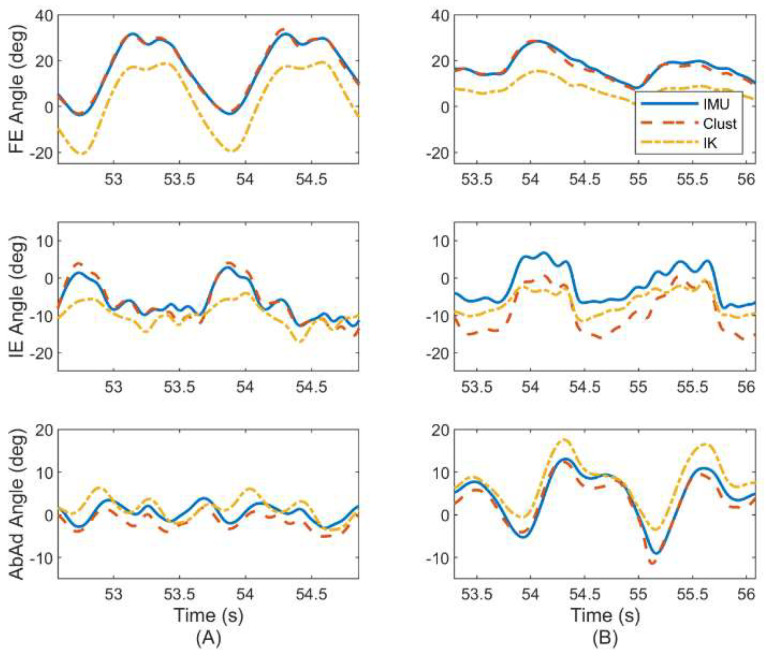
Representative hip joint angle estimates over two stride cycles for two different gaits. Hip joint angle estimates for normal treadmill walking (**A**) and lateral left walking (**B**). Estimates are made utilizing ErKF (IMU), cluster (Clust), and inverse kinematics (IK) methods.

Finally, we examine the performance of the ErKF method for the other types of gait included in the study. Figure 10 illustrates a side-by-side comparison of the left hip joint angle trajectory estimates for (Figure 10A) normal treadmill walking and (Figure 10B) walking laterally left using all three estimation methods (ErKF, Clust, IK) for the same representative subject. As described above, these two gaits induce limiting case motions of the hip with normal walking inducing predominantly hip FE and lateral walking inducing predominantly hip AbAd. The left hip is chosen for this subject (same considered above) to evaluate the method without the confounding effects of likely sensor to segment alignment errors for the right hip (refer to Section 4.6, “Factors leading to abnormally poor estimates”). Refer to Appendix A for example joint angle estimates over two stride cycles for all six gait types for the same representative subject.

## 4. Discussion

This paper extends the ErKF method developed in [37] for a three-body mechanical walker to a full seven-body model of the human lower limbs and then evaluates its performance across a large range of gait types. The results demonstrate that the ErKF method estimates important kinematic parameters comparable to those estimated using two different MOCAP methods across six different walking gaits.

### 4.1. ErKF Estimates of Instantaneous Joint Angles

In general, the method yields small mean RMS differences (<5 deg in 94% of the cases) in estimates of FE and AbAd joint angles (DP and InEv for the ankle) across all joints and gait types with low offsets when compared to the cluster method (refer to Table 2). Higher offsets typically occur in IE joint angle estimates; however, these higher IE offsets vary greatly between subjects as evidenced by the high standard deviations reported in Table 2 for IE angles compared to the other joint angles. Additionally, these higher IE offsets generally stabilize over the one-minute trials to near-constant values with the remaining joint angle waveform well-estimated; see, for example, Figure 9. Importantly, the differences in range of motion estimates remain small (generally <5 deg) between the ErKF and the MOCAP methods, even for the IE angles for the hips and knees (refer to Table 4 and Table 5). While RMS differences in IE joint angle estimates are generally higher than the other degrees of freedom (refer to Table 2), this trend appears typical of IMU-based methods for the lower limbs, perhaps due to low signal-to-noise ratio in the transverse plane kinematics [18]. Thus, researchers should exercise caution when interpreting IE estimates obtained from IMU-based methods (including the method presented in this paper), especially when RMS differences in these estimates are large compared to the expected ranges of motion. The differences in estimates of the joint angle between the ErKF and inverse kinematics (MOCAP) methods are generally greater than those between the ErKF and cluster methods (refer to Table 3). This likely arises from several factors including the movement of the underlying bones relative to the sensors/markers.

### 4.2. ErKF Estimates of Stride Parameters

Mean RMS differences in stride length remain below 0.08 m for normal, fast, and both lateral walking gaits with mean RMS differences in step width of 0.05 m for normal and fast walking (refer to Table 6). Importantly, recall that MOCAP estimates of stride length in this study rely on accurate estimates of belt speed. While not reported here, we observe that differences between belt speeds estimated via MOCAP (as used in this paper) and those reported by the treadmill (which were not available for all trials) are often on the order of 0.03 m/s. Thus, errors in MOCAP-estimated belt speed may account for a significant portion of differences in estimated stride length between the ErKF method and MOCAP in this study. Additionally, we observe that trials with low offsets in joint angles typically exhibited smaller differences in stride metric estimates. Thus, similar error sources may be responsible for poor stride metric and joint angle estimates. Such error sources include soft tissue artefacts, sensor to segment alignment errors, MOCAP errors, and/or the imposition of the hinge-like joint measurement.

### 4.3. Utility of ErKF Joint Angle Range of Motion Estimates

In addition to comparing joint angle estimates, we also compare joint angle range of motion estimates between the ErKF and MOCAP methods. Range of motion, and particularly changes in range of motion, have demonstrated significance in biomechanical studies in a variety of contexts as we emphasize by citing four examples. Devita and Hortobagyi [64] evaluate hip and ankle flexion range of motion during stance for walking and find significant differences between elderly and young populations (3.3 deg for hip and −2.7 for ankle, elderly-young). Qu and Yeo [55] observe that fatigue ultimately increases the sagittal-plane hip and knee range of motion by an average of 1.3 and 1.9 deg, respectively, in their study. Sofuwa et al. [65] examine differences between cohorts with and without Parkinsons’s disease and conclude that the “healthy” cohort exhibits 4.8 and 4.0 deg greater ankle DP range of motion during the push-off and swing phases, respectively. Carmo et al. [61] contrast post-stroke patients and healthy controls and find that knee flexion range of motion is 17.4 and 20.0 degrees lower for stroke patient’s affected side compared to their unaffected side and healthy controls, respectively. These example studies highlight the important need to measure joint range of motion for biomechanical studies. Consequently, we anticipate that the ErKF method may prove valuable for future studies, and particularly if one can also establish the measurement resolution of range of motion and changes in range of motion. While the present study was not designed to establish measurement resolution, the favorable comparisons across the methods may indeed suggest sufficient resolution for biomechanical studies as explained next.

The ErKF method demonstrates average RMS differences for hip FE, IE, and AbAd range of motion less than 2, 3, and 4 deg, respectively compared to the cluster method during normal walking (refer to Table 4; recall cluster estimates of range of motion are not available for comparison on the knees and ankles due to frequent marker occlusion). Importantly, note that range of motion can often be estimated similarly between ErKF and both MOCAP methods even in the presence of systematic offsets between the ErKF- and MOCAP-estimated joint angle waveforms (see for example, Figure 9 and Figure 10). Further note that these waveform estimates are more commonly similar in joint degrees of freedom that dominate a particular gait (for example, hip FE for normal walking, hip AbAd for lateral walking; refer to Figure 10). Comparing the ErKF method to the inverse kinematics method, range of motion differences are slightly higher than compared to the cluster method, but still generally below 5 degrees across joints and gait types (refer to Table 5). However, note that the generally larger differences in Table 5 (comparison to inverse kinematics method) versus those of Table 4 (comparison to cluster method) may result from differences between the two MOCAP-based methods themselves (i.e., offsets between Clust and IK estimates; refer to Figure 10). Additionally, the larger differences in ranges of motion for the ankle angles versus those for the knee and hip may derive from increased complexity of the ankle joint in the Gait2354 model used for the IK estimates versus the simpler model used in the ErKF and cluster methods. Nevertheless, the low RMS differences in range of motion estimates between the ErKF method and MOCAP methods (Table 4 and Table 5) establish that the ErKF method yields very similar estimates of range of motion compared to MOCAP. Importantly, note also that the differences between the ErKF and MOCAP methods in this study are similar to and often smaller than the changes in range of motion observed in the studies highlighted above. This fact supports the claim that the ErKF method may possess sufficient resolution in range of motion estimates to support meaningful biomechanical studies outside (and within) the laboratory.

Further, in clinical settings, typically specific mean ranges of motion (i.e., averaged across a trial) are of greater interest than stride-by-stride changes in range of motion. Thus, Bland–Altman plots are used to evaluate the agreement between ErKF and MOCAP estimates of mean range of motion for two exemplary joint angles, namely the left knee FE angle (Figure 5) and the left hip AbAd angle (Figure 6). These plots demonstrate that the ErKF method agrees well with the inverse kinematics method for estimates of mean range of motion for some joint angles across two very different gait types (normal walking verses lateral left walking). These results motivate future investigation into clinical applications where the ErKF method may prove valid.

### 4.4. Comparison of ErKF Method to other IMU-Based Methods

The differences between ErKF and MOCAP-based estimates of joint angles and ranges of motion in this study are comparable to prior IMU-based methods that focused on normal walking, as detailed below. Importantly though, the present method advances well beyond the prior methods in: (1) estimating three-dimensional joint angles across all lower-limb joints, (2) succeeding over a wide variety of gait types (beyond normal walking), and/or (3) eliminating reliance on prior assumptions (e.g., clean magnetic field, movement assumptions). For example, Adamowicz et al. [29], who employ an overlapping data set with that used in this study, present a method specific for the hip joint and observe mean RMS differences in hip FE, IE, and AbAd of 8.6, 10.0, and 8.0 deg, respectively during normal walking when compared to a similar cluster-based MOCAP method. The present ErKF method demonstrates superior mean RMS differences for hip FE, IE, and AbAd of 2.4, 7.4, and 3.8 deg, respectively for normal walking (refer to Table 2). However, caution should be exercised when comparing these results since the method of Adamowicz et al. is an “IMU-only” method versus the present study which leverages MOCAP-based initializations. Weygers et al. [24] develop a method specific for the knee joint and observe RMS differences from MOCAP less than 5 deg during walking for knee Euler angles (as opposed to anatomical angles). While acknowledging the differences in Euler angles versus anatomical angles, similar differences (<5 deg) arise in the ErKF method for the knee during normal walking for FE and AbAd, but not for IE (6.6 deg); refer to Table 3. Importantly, these prior studies offer “single-joint” (two-body) methods as opposed to the multi-joint (seven-body) method developed herein.

The present ErKF method also compares well with prior multi-joint methods and also removes assumptions and/or reliance on magnetometer data. Teufl et al. develop a seven-body model of the lower limbs and compare IMU-derived results with those from MOCAP using both cluster and inverse kinematics methods for normal speed overground walking [22,56] and for short dynamic movements [28]. For normal walking, they observe smaller RMS differences in joint angles than reported in this study. For example, in [22] they observe RMS differences in FE (or DP), IE, and AbAd (or InEv) across all joints up to 1.6, 2.3, and 1.6 deg, respectively compared to a similar cluster method and up to 5.4, 5.5, and 4.2 deg, respectively compared to a MOCAP method relying on bony landmarks. In the current study, the analogous RMS differences are up to 4.2, 8.8, and 4.6, deg, respectively compared to the cluster method (Table 2) and up to 7.2, 8.6, and 4.7 deg, respectively compared to the inverse kinematics method which primarily relies on bony landmarks (Table 3). They also report stride length and step width estimates during walking [56], having RMS differences of 0.04 m and 0.03 m, respectively compared to 0.07 and 0.05 m, respectively in this study; refer to Table 6. Note that in [22,56], Teufl et al. studied overground walking and thus their results are unaffected by errors in MOCAP-estimated belt speed encountered herein. Additionally, their method relies critically on a level ground assumption (for both footfall identification and to correct drift errors) which is not assumed in the present ErKF method. Thus, while the present method can be used on level or uneven terrain (i.e., outdoor environments), the method of [22] is restricted to level terrain (i.e., likely restricted to indoor, single-level environments). Additionally, the process model in [22] assumes constant linear acceleration and angular velocity (with IMU measurements being used in the measurement model) and thus it may not be suited for more dynamic movements. Finally, their method is not evaluated for any gait types other than normal speed walking in contrast to this study that examines six types of gait. McGrath and Stirling [23] develop a seven-body method for lower-limb kinematic estimation. However, they evaluate it solely for knee FE yielding mean RMS differences of 4.3 deg compared to a MOCAP-based inverse kinematics method and using a specific set of calibration motions designed to excite all lower-limb degrees of freedom (i.e., different than the walking gaits in this paper for the ErKF method). As shown in Table 3, the ErKF method yields similar differences with MOCAP for the knee FE (4.1 deg), although for a different set of movements. Zhang et al. [66] validate the performance of a commercial Xsens system for walking and stair ascent/descent. They report mean and standard deviation joint angle differences up to 5.1 and 4.2 degrees, respectively for walking (depending on the joint). However, they do not report RMS differences and so direct comparisons cannot be made to this study. Nüesch et al. [67] validate the commercial RehaGait system for treadmill walking and report RMS differences of 9.6, 7.6, and 4.5 deg for FE of the hip, knee, and ankle, respectively. Compared to this study, the ErKF method demonstrates superior or comparable FE estimates for all joints with RMS differences of 7.1, 4.1, and 5.3 deg for the hip, knee, and ankle, respectively (refer to Table 3). As a further distinction, we also note that commercial systems generally also employ magnetometer data (not used for the ErKF) and rely on proprietary algorithms; thus, it is difficult to evaluate their limitations.

### 4.5. Performance of Novel Hip “Soft” Hinge Correction

Recall that an important contribution of the present ErKF method is the novel “soft” hinge correction applied to the hip at all time points (but with a large measurement uncertainty to acknowledge that the hip is often not acting as a pure hinge) to aid in constraining orientation integration drift. In applying this correction, it is critical to assess whether the ErKF yields accurate hip joint angle estimates, particularly the hip AbAd angle for movements where the hip manifestly does not act as a hinge (e.g., lateral walking). In Table 2, Table 3, Table 4 and Table 5, we do not observe any clear degradation in estimates of the instantaneous hip AbAd angle or its range of motion between normal walking (predominantly FE) and lateral walking (predominantly AbAd). Further, in Figure 10, observe the remarkably close agreement between the ErKF and cluster estimates of the left hip joint angles for both normal walking and lateral left walking for a representative subject, especially for FE and AbAd angles. Finally, Figure 6 shows Bland–Altman plots of the mean left hip AbAd range of motion over a trial for normal walking (Figure 6A) and lateral left walking (Figure 6B). Notice the close agreement in these estimates for both movement conditions. These results confirm the success of the novel hip soft hinge constraint as the hip joint angles are estimated consistently between the methods even for lateral walking gait where the hip rotation is dominated by AdAd and not FE.

### 4.6. Factors Leading to Abnormally Poor Estimates

While MOCAP data are used in the present ErKF method in part to reduce the risk of obtaining poor sensor to segment alignment parameters and initializations, we emphasize that MOCAP does not yield ground truth estimates. Thus, large differences between ErKF and MOCAP estimates may be the results of errors in either (or both) method types. Figure 8 provides evidence that errors in the sensor to segment alignment (obtained using MOCAP data) can significantly impact estimated joint angles; however, the ErKF method is capable of overcoming such errors in estimating several key metrics. We arrive at this conclusion from the following observations. For the left hip (A), observe the excellent agreement between the ErKF and cluster method for all three joint angles over the entire trial (as expected from Figure 7). However, for this very same subject and trial, the right hip (Figure 8B) exhibits an offset between the two methods. This offset develops when the joint center measurement is utilized for a trial and for two likely reasons. First, this offset is influenced by the joint center measurement which relies on accurate estimates of the joint center locations in the IMU frames (obtained from the sensor to segment alignment) to accurately enforce the kinematic constraints. Second, given that this offset can vary between the hip joints for the same subject and trial, the offset likely originates from errors in the sensor to segment alignment parameters (determined using MOCAP data) as they are distinct for the two hip joints. To further support this claim (i.e., that the observable differences likely result from MOCAP-aided sensor to segment alignment rather than the ErKF method), note that the MOCAP-based FE estimates for the right hip appear to be qualitatively incorrect because they obviously oscillate between different extremes than the left hip and the peak extension for each stride (local minima in FE) is much larger than expected for healthy subjects in normal walking [8]. These obvious errors likely occur from errors in marker placement and model scaling assumptions, thus resulting in sensor to segment alignment errors. However, because no sensor to segment alignment method is immune to errors, it is critical that the ErKF method still provide meaningful estimates even in the presence of such errors. Thus, we do not exclude any trials from our analysis for obvious errors in sensor to segment alignment. Additionally, observe that despite these errors, estimates from the ErKF method do not rapidly diverge like estimates using raw integration (refer to Figure 7). Thus, errors in sensor to segment alignment may still lead to converged estimates, only with systematic offsets between methods due to the errors in sensor to segment alignment which affect both ErKF and MOCAP estimates (albeit affecting the two methods differently). In rare cases, the differences in the joint angle waveforms between different methods did not converge to a steady offset over the one-minute trials. However, we observed these cases of larger drift arise for specific subjects rather than to gait types, further indicating that poor sensor to segment alignment may be primarily responsible.

We duly note that a minority of the kinematic estimates in this study are poorer for specific gaits. For example, while we observe overall excellent estimates of ankle, knee, and hip joint angles across gait types, the hip IE estimates exhibit much higher differences for slow, backward, and lateral walking than for the faster (>0.8 m/s) forward walking trials (refer to Table 2 and Table 3). We observe this same trend for estimates of stride length and step width (i.e., smaller differences for the two faster forward walks than for the other gaits; refer to Table 6). However, while not reported here, we also observe that these cases of larger mean RMS differences for specific gaits are largely attributed to certain subjects who consistently exhibited higher differences in estimated joint angles and stride metrics across gait types compared to others. This suggests that subject-specific systematic errors (i.e., in sensor to segment alignment, marker placement, joint center locations) may be the primary cause behind these rare, but poorer, kinematic estimates. Thus, better methods for sensor to segment alignment may yield significant improvements to the results presented here. Finally, we note the poorer stride metric estimates for slow walking compared to the other gaits; refer to Table 6. However, this gait is much slower (<0.5 m/s) than is typical of most populations and the larger differences may be associated with lower signal-to-noise ratios in the IMU data (assuming the same sensor hardware selection). Despite the exceptions duly noted above, the ErKF method provides kinematic estimates that closely replicate those from MOCAP and across a broad range of gait types. Additionally, the method does not rely on “laboratory-like” assumptions (e.g., level ground) nor does it rely on magnetometer data (susceptible to pollution by magnetic interferences in both indoor and outdoor environments)

## 5. Limitations

Several limitations also exist with the current method. First, MOCAP data are used to determine sensor to segment alignment; thus, it is not yet an “IMU-only” method. However, determining sensor to segment alignment for wearable IMUs represents a major topic of research in itself [38,39,40] and promising existing methods could be incorporated in the ErKF method to yield an IMU-only method. Another promising solution is to expand the ErKF method to a “self-calibrating” method that estimates the sensor to segment alignment parameters simultaneously with the kinematic estimates (i.e., as part of the full state). As described in Section 4, while this study does not assume laboratory-like conditions (e.g., level ground), the method is evaluated only on data from treadmill walking trials. Future studies should examine the accuracy of the method on uneven terrain including outdoor environments. In the present study, trials are only one minute in length, and future work should evaluate the method on longer trials to ensure that drift errors remain constrained over long times (e.g., hours). The accuracies of joint angle estimates are compared to two different MOCAP-based methods to evaluate the performance of the ErKF method. Comparison against the MOCAP cluster method enables evaluation of the ErKF method without some of the soft tissue artefacts. However, we emphasize that errors in sensor to segment alignment parameters (including those due to marker misplacement and inaccurate joint center location estimates) and movement of the IMU relative to the underlying bone all affect the method. Thus, errors in MOCAP marker placement will affect all three estimation methods used in this study. Similarly, the current methods rely on static estimates of joint centers (i.e., estimation from marker locations and anthropometrics). These methods rely on new subjects having similar characteristics to those used to determine the estimation equations and then on accurate marker placement (as emphasized above). While the present study demonstrates good accuracy for select kinematic metrics, future studies should evaluate accuracy for other relevant biomechanical metrics (e.g., segment velocities). Finally, note that comparisons between this study and other methods are inherently difficult due to a multitude of differences in experimental hardware and procedures including in marker placement, recruited subject populations, MOCAP reference systems used, IMU hardware selection (see [26]), tasks performed, study design, and data processing techniques. Thus, future comparison of filtering methods alone will require commonly shared data sets, including those containing highly accurate ground truth data for reference.

## 6. Conclusions

In this study, a novel ErKF method is extended to a full (seven-body) model of the lower limbs for human subjects. Doing so brings new challenges including: (1) increasing the degrees of freedom, (2) characterizing complex biological (versus mechanical) joints (e.g., joint center location and sensor to segment alignment), and (3) soft tissue artefacts. Importantly, this paper contributes a novel application of the joint axis measurement correction in the ErKF for the hip and knee to reduce angle drift errors. In contrast to previous IMU-based joint axis correction methods (specific to the knee), this new method reduces drift errors without assuming strict hinge-like behavior during certain times. Thus, the correction herein is also applicable to the hip (but with different measurement noise parameters). Significantly, this work validates the ErKF method on human subjects walking with six different gait types including forward walking (at slow, normal, and fast speeds), backward walking, and lateral walking (both left and right). The method’s demonstrated agreement (compared to MOCAP) in estimating joint angles, joint angle range of motion, stride length, and step width across all six gait types studied with healthy subjects (including forced abnormal gaits) motivates its future evaluation on subjects with gait abnormalities (e.g., due to injury and/or disease). In particular, for all gait types studied, RMS differences between ErKF and MOCAP cluster-based joint angle estimates generally remain below 5 degrees for all three ankle joint angles and for flexion/extension and abduction/adduction of the hips and knees. Additionally, RMS differences between ErKF and MOCAP inverse kinematics estimates in (stride to stride) range of motion generally remain below 5 degrees for all hip and knee joint angles (with slightly higher differences for the ankle joint angles) and across all gait types. Finally, mean RMS differences between ErKF and MOCAP estimates for both stride length and step width remain below 0.13 m across all gait types (except stride length for slow forward walking) and below 0.07 m for the two fastest walking gaits (>0.8 m/s). The overall comparability between estimates obtained using the ErKF method and MOCAP confirm the significant promise for using the ErKF method for non-laboratory based biomechanical studies of the human lower limbs in broad contexts.

## Figures and Tables

**Table 1:** IMU and measurement noise values for the ErKF method. The noise values for the accelerometer $\sigma_{a}$ and gyroscope $\sigma_{\omega }$ in the process model. The noise values for the zero-velocity $\sigma_{ZV}$, gravitational tilt $\sigma_{tilt}$, joint center $\sigma_{JC}$, knee joint axis $\sigma_{JA,Knee}$, and hip joint axis $\sigma_{JA,Hip}$ in the measurement models.

Noise Parameter	$\sigma_{a}$ (m/s^2^)	$\sigma_{\omega }$ (deg/s)	$\sigma_{ZV}$ (m/s)	$\sigma_{tilt}$ (deg)	$\sigma_{JC}$ (m)	$\sigma_{JA,Knee}$ (deg)	$\sigma_{JA,Hip}$ (deg)
Value	0.013	2.83	0.01	5.73	0.01	1.15	57.3

**Table 2:** Comparisons of joint angles with Cluster Method. Mean (Standard Deviation) of RMS difference (IMU-Clust) in estimates for each joint angle. All angles in degrees. Green, yellow, and red highlights indicate mean values <5 deg, <10 deg, and ≥10 deg, respectively. Results segmented by type of walking gait; namely normal forward (Normal), fast forward (Fast), slow forward (Slow), backward (Back), lateral left (Left), and lateral right (Right). Joint angles are flexion/extension (FE), internal/external rotation (IE), abduction/adduction (AbAd), dorsiflexion/plantarflexion (DP), and inversion/eversion (InEv). R and L prepended to joint names indicate right and left side, respectively.

	Normal	Fast	Slow	Back	Left	Right
RHip FE	2.54 (1.23)	2.28 (0.86)	2.21 (0.80)	2.34 (1.18)	2.80 (1.89)	2.94 (1.94)
LHip FE	2.17 (0.89)	2.25 (0.83)	2.40 (1.44)	2.61 (1.22)	3.00 (1.61)	2.63 (1.41)
RKnee FE	3.13 (1.06)	3.37 (0.83)	3.07 (1.21)	3.10 (1.13)	3.77 (1.96)	3.65 (1.78)
LKnee FE	3.16 (1.05)	3.52 (1.37)	2.99 (1.17)	3.07 (1.33)	3.71 (1.13)	3.05 (1.18)
RAnkle DP	4.24 (1.81)	3.99 (1.70)	5.03 (2.04)	5.50 (1.80)	6.32 (1.98)	6.00 (1.89)
LAnkle DP	3.00 (1.23)	3.13 (1.15)	3.41 (1.22)	2.87 (1.51)	3.31 (1.63)	3.02 (1.53)
RHip IE	7.40 (3.56)	6.08 (3.00)	7.40 (5.75)	10.55 (9.44)	8.30 (9.52)	9.44 (8.45)
LHip IE	7.47 (7.60)	6.57 (4.68)	11.97 (12.76)	11.45 (10.89)	10.35 (7.83)	8.17 (7.89)
RKnee IE	8.83 (3.88)	7.96 (4.00)	8.21 (3.60)	8.48 (2.95)	6.87 (3.28)	7.34 (3.47)
LKnee IE	7.29 (6.96)	6.74 (6.59)	7.63 (8.05)	8.29 (7.27)	9.83 (8.76)	9.38 (8.12)
RAnkle IE	2.72 (1.10)	2.40 (0.85)	3.83 (1.90)	2.81 (1.38)	3.74 (1.82)	4.15 (1.79)
LAnkle IE	3.14 (1.76)	2.58 (1.35)	3.85 (1.75)	3.10 (1.75)	3.19 (1.95)	3.29 (1.77)
RHip AbAd	4.15 (2.13)	3.49 (1.69)	3.52 (2.25)	3.85 (3.02)	3.43 (2.70)	4.04 (3.36)
LHip AbAd	3.45 (2.05)	3.40 (1.66)	4.07 (2.32)	3.67 (1.76)	4.09 (1.93)	2.92 (1.28)
RKnee AbAd	4.62 (3.40)	4.52 (3.36)	4.24 (3.70)	3.38 (3.40)	3.16 (2.91)	3.64 (3.54)
LKnee AbAd	4.60 (1.68)	4.46 (1.84)	4.31 (1.64)	3.85 (1.74)	3.62 (1.56)	3.62 (1.64)
RAnkle InEv	2.61 (0.79)	2.72 (0.80)	2.70 (1.00)	1.96 (0.68)	2.42 (0.66)	2.58 (0.67)
LAnkle InEv	2.40 (0.94)	2.35 (1.16)	2.51 (0.88)	2.00 (1.07)	2.08 (0.68)	2.27 (0.89)

**Table 3:** Comparisons of joint angles with Inverse Kinematics Method. Mean (Standard Deviation) of RMS difference (IMU-IK) in estimates for each joint angle. All angles in degrees. Green, yellow, and red highlights indicate mean values <5 deg, <10 deg, and ≥10 deg, respectively. Results segmented by type of walking gait; namely normal forward (Normal), fast forward (Fast), slow forward (Slow), backward (Back), lateral left (Left), and lateral right (Right). Joint angles are flexion/extension (FE), internal/external rotation (IE), abduction/adduction (AbAd), dorsiflexion/plantarflexion (DP), and inversion/eversion (InEv). R and L prepended to joint names indicate right and left side, respectively.

	Normal	Fast	Slow	Back	Left	Right
RHip FE	7.03 (4.37)	7.16 (4.21)	5.96 (4.49)	6.33 (4.53)	5.67 (4.52)	6.03 (4.73)
LHip FE	7.23 (4.03)	7.00 (3.71)	6.75 (3.85)	6.97 (4.55)	6.33 (4.09)	6.73 (4.46)
RKnee FE	4.36 (2.14)	4.74 (2.10)	4.16 (2.26)	4.35 (1.89)	4.50 (2.14)	4.64 (2.09)
LKnee FE	3.79 (1.26)	4.14 (1.26)	3.37 (1.54)	4.18 (1.48)	4.15 (1.63)	3.84 (1.70)
RAnkle DP	5.41 (2.98)	5.48 (3.07)	5.40 (2.92)	5.20 (2.64)	4.33 (2.46)	4.92 (2.61)
LAnkle DP	5.28 (2.14)	5.64 (2.22)	4.76 (2.35)	3.52 (1.87)	4.62 (4.13)	3.59 (1.77)
RHip IE	7.43 (3.59)	6.19 (2.55)	8.50 (6.43)	11.26 (10.24)	9.58 (8.54)	11.03 (9.44)
LHip IE	8.56 (7.43)	7.21 (4.95)	13.54 (13.34)	12.67 (11.30)	11.61 (9.19)	9.28 (8.41)
RKnee IE	5.91 (2.71)	5.66 (2.34)	5.98 (3.01)	6.12 (3.30)	5.81 (3.27)	5.83 (3.08)
LKnee IE	7.32 (5.20)	7.70 (5.11)	6.87 (5.93)	7.43 (5.94)	8.62 (6.79)	7.66 (6.01)
RAnkle IE	4.23 (2.32)	4.49 (1.28)	4.39 (2.06)	3.90 (2.49)	4.81 (3.30)	3.89 (2.55)
LAnkle IE	4.76 (2.68)	5.35 (2.72)	4.49 (2.43)	3.73 (2.30)	4.87 (3.93)	4.17 (1.82)
RHip AbAd	4.74 (2.04)	4.53 (1.77)	4.33 (1.35)	3.88 (1.96)	3.39 (1.61)	4.51 (1.79)
LHip AbAd	4.37 (1.47)	4.44 (1.47)	4.64 (1.78)	4.00 (1.41)	4.27 (1.65)	3.52 (1.63)
RKnee AbAd	3.99 (1.53)	4.26 (1.66)	3.17 (1.22)	2.65 (1.28)	2.65 (1.63)	2.67 (1.35)
LKnee AbAd	3.90 (2.06)	3.72 (1.81)	3.26 (1.88)	3.73 (1.97)	2.96 (1.61)	3.55 (2.06)
RAnkle InEv	4.56 (1.32)	4.94 (1.51)	4.88 (1.32)	5.79 (1.57)	6.27 (1.22)	6.07 (1.80)
LAnkle InEv	4.70 (1.50)	5.16 (1.72)	4.42 (1.18)	4.71 (1.68)	5.35 (1.89)	5.48 (2.11)

**Table 4:** Comparisons of range of motion with Cluster Method. Mean (Standard Deviation) of RMS difference (IMU-Clust) in range of motion estimates. All angles in degrees. Green highlights indicate mean values <5 deg. No results reported for knees or ankles because frequent marker occlusion prevented Cluster estimates. Results segmented by type of walking gait; namely normal forward (Normal), fast forward (Fast), slow forward (Slow), backward (Back), lateral left (Left), and lateral right (Right). Joint angles are flexion/extension (FE), internal/external rotation (IE), abduction/adduction (AbAd), dorsiflexion/plantarflexion (DP), and inversion/eversion (InEv). R and L prepended to joint names indicate right and left side, respectively. Minimum of 10 subjects represented per entry.

	Normal	Fast	Slow	Back	Left	Right
RHip FE	1.22 (0.36)	1.60 (0.69)	1.35 (0.57)	2.26 (0.85)	2.39 (1.89)	1.58 (0.58)
LHip FE	1.69 (1.54)	1.69 (1.32)	3.59 (4.82)	2.74 (2.31)	2.19 (1.23)	1.86 (1.20)
RHip IE	1.98 (0.99)	1.98 (0.71)	2.41 (0.87)	2.53 (1.06)	3.28 (1.60)	3.22 (1.53)
LHip IE	2.15 (0.80)	2.10 (0.64)	2.41 (0.92)	3.15 (1.04)	2.92 (1.22)	2.54 (1.04)
RHip AbAd	3.14 (1.84)	2.98 (2.08)	2.51 (1.61)	2.27 (0.98)	2.97 (1.22)	2.26 (0.96)
LHip AbAd	3.29 (3.19)	2.24 (1.75)	4.24 (4.99)	3.02 (3.27)	2.58 (1.97)	2.77 (1.21)

**Table 5:** Comparisons of range of motion with Inverse Kinematics Method. Mean (Standard Deviation) of RMS difference (IMU-IK) in range of motion estimates. All angles in degrees. Green, yellow, and red highlights indicate mean values < 5 deg, <10 deg, and ≥10 deg, respectively. Results segmented by type of walking gait; namely normal forward (Normal), fast forward (Fast), slow forward (Slow), backward (Back), lateral left (Left), and lateral right (Right). Joint angles are flexion/extension (FE), internal/external rotation (IE), abduction/adduction (AbAd), dorsiflexion/plantarflexion (DP), and inversion/eversion (InEv). R and L prepended to joint names indicate right and left side, respectively.

	Normal	Fast	Slow	Back	Left	Right
RHip FE	4.17 (2.40)	4.61 (2.72)	3.26 (1.85)	3.70 (2.38)	2.13 (0.88)	2.44 (0.90)
LHip FE	4.14 (2.07)	4.56 (2.33)	4.59 (4.25)	2.94 (1.99)	2.33 (0.99)	2.21 (1.02)
RHip IE	5.22 (2.75)	5.53 (2.50)	4.84 (2.89)	4.05 (2.17)	5.92 (3.58)	4.20 (2.26)
LHip IE	4.77 (2.71)	5.28 (2.21)	4.49 (2.77)	4.13 (2.42)	3.69 (1.43)	5.93 (2.82)
RHip AbAd	3.48 (2.20)	4.40 (2.66)	3.29 (2.30)	3.28 (1.58)	2.43 (1.51)	2.36 (1.45)
LHip AbAd	4.30 (1.88)	5.71 (2.42)	4.41 (2.42)	4.00 (2.43)	2.74 (2.40)	2.69 (1.39)
RKnee FE	4.62 (2.65)	4.37 (2.65)	4.53 (2.71)	3.01 (1.88)	2.94 (1.84)	2.88 (1.08)
LKnee FE	3.44 (2.22)	2.89 (2.18)	3.61 (2.12)	2.92 (1.50)	3.14 (1.10)	2.57 (0.98)
RKneeIE	4.88 (2.60)	5.43 (3.41)	3.14 (2.15)	3.61 (1.74)	4.21 (1.44)	3.90 (1.61)
LKnee IE	4.71 (2.05)	5.16 (1.74)	3.16 (1.43)	3.28 (1.39)	4.85 (2.06)	4.61 (1.64)
RKnee AbAd	5.77 (4.36)	6.85 (5.11)	4.00 (2.66)	2.35 (1.52)	1.51 (0.87)	1.89 (0.80)
LKnee AbAd	3.70 (3.01)	3.81 (2.69)	3.66 (2.67)	2.71 (1.47)	2.05 (1.09)	1.99 (0.82)
RAnkle DP	4.49 (2.29)	5.38 (2.26)	3.63 (2.10)	2.50 (1.23)	2.33 (0.65)	2.51 (1.54)
LAnkle DP	5.26 (2.80)	6.21 (2.93)	3.05 (1.71)	1.84 (0.87)	5.36 (11.45)	2.14 (1.07)
RAnkle IE	8.21 (3.18)	9.33 (2.78)	6.71 (2.77)	4.05 (1.39)	3.71 (1.59)	3.15 (1.81)
LAnkle IE	8.90 (3.89)	10.57 (3.05)	6.86 (3.01)	3.33 (1.74)	5.69 (11.62)	2.76 (1.76)
RAnkle InEv	3.79 (1.72)	3.22 (1.16)	4.89 (1.99)	5.65 (2.49)	6.10 (2.73)	7.21 (3.22)
LAnkle InEv	5.27 (2.75)	4.52 (2.57)	5.77 (3.27)	5.62 (2.45)	7.57 (3.45)	5.66 (2.31)

**Table 6:** Comparisons of stride parameters with MOCAP. Mean (standard deviation) of RMS difference (IMU-MOCAP) in estimates of stride length (SL) and step width (SW). Additionally, mean (standard deviation) of the mean SL, SW, and belt speed (all estimated by MOCAP) across all subjects. Finally, mean RMS differences as percentage of the mean SL and SW (% Diff). All distances in m, speeds in m/s. Results segmented by type of walking gait; namely normal forward (Normal), fast forward (Fast), slow forward (Slow), backward (Back), lateral left (Left), and lateral right (Right). NA indicates not applicable because mean value near zero.

	Normal	Fast	Slow	Back	Left	Right
SL RMS Diff	0.07 (0.03)	0.05 (0.02)	0.16 (0.05)	0.11 (0.05)	0.07 (0.03)	0.06 (0.02)
Mean SL	1.09 (0.15)	1.40 (0.17)	0.84 (0.15)	0.62 (0.15)	0.48 (0.08)	0.46 (0.08)
% Diff SL	6.0%	3.4%	19.1%	17.6%	15.5%	13.3%
SW RMS Diff	0.05 (0.02)	0.05 (0.02)	0.07 (0.04)	0.12 (0.06)	0.10 (0.08)	0.09 (0.05)
Mean SW	0.12 (0.03)	0.11 (0.03)	0.13 (0.03)	0.15 (0.03)	0.01 (0.02)	0.00 (0.02)
% Diff SW	40.4%	43.1%	57.4%	80.8%	NA	NA
Belt Speed	0.86 (0.17)	1.32 (0.21)	0.47 (0.09)	0.43 (0.08)	0.39 (0.07)	0.38 (0.08)

**Figure A1:**
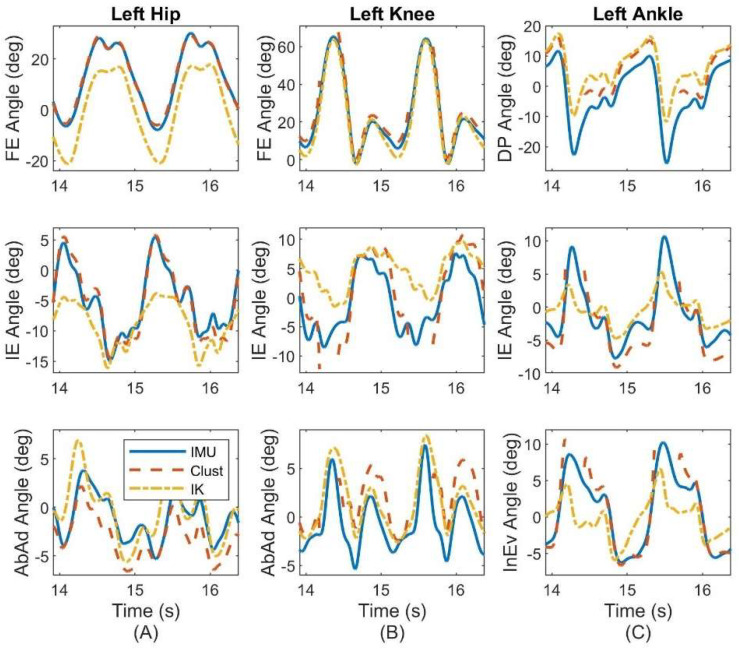
Normal Walking. Example joint angle estimates over two stride cycles for normal treadmill walking. Plots show the three anatomical angles for (**A**) left hip, (**B**) left knee, and (**C**) left ankle. Estimates are also compared between ErKF (IMU), cluster (Clust), and inverse kinematics (IK) methods.

**Figure A2:**
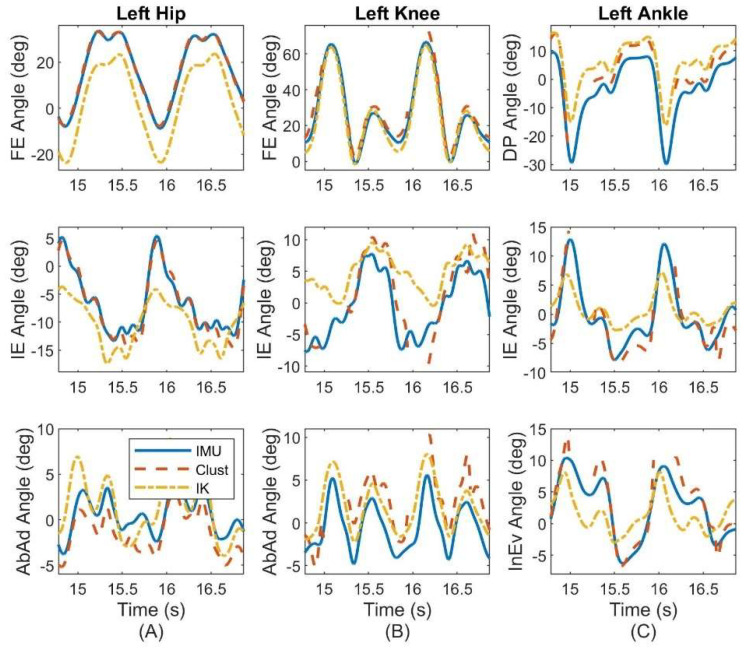
Fast Walking. Example joint angle estimates over two stride cycles for fast treadmill walking. Plots show the three anatomical angles for (**A**) left hip, (**B**) left knee, and (**C**) left ankle. Estimates are also compared between ErKF (IMU), cluster (Clust), and inverse kinematics (IK) methods.

**Figure A3:**
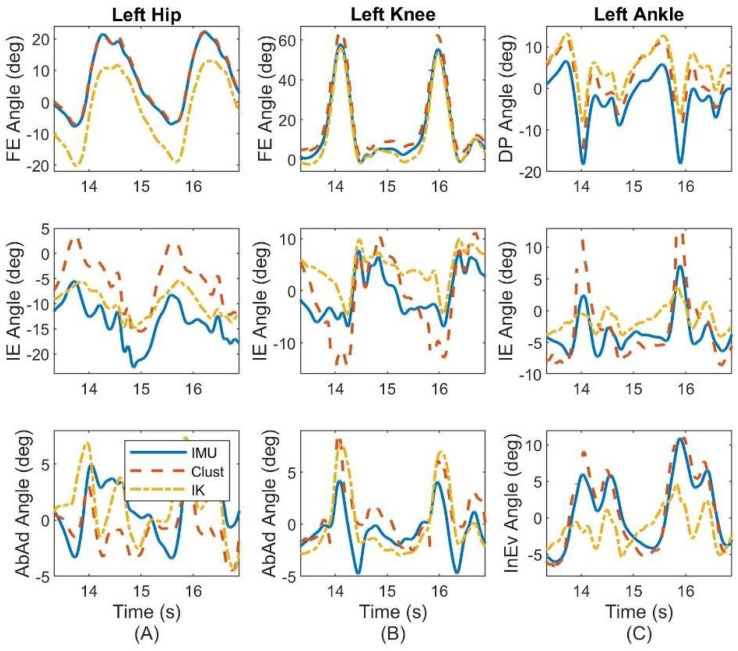
Slow Walking. Example joint angle estimates over two stride cycles for slow treadmill walking. Plots show the three anatomical angles for (**A**) left hip, (**B**) left knee, and (**C**) left ankle. Estimates are also compared between ErKF (IMU), cluster (Clust), and inverse kinematics (IK) methods.

**Figure A4:**
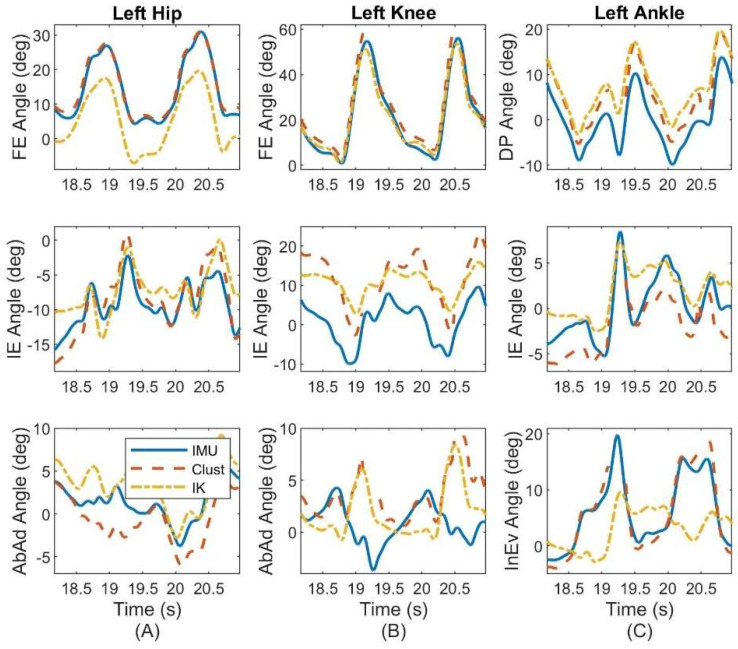
Backward Walking. Example joint angle estimates over two stride cycles for backward treadmill walking. Plots show the three anatomical angles for (**A**) left hip, (**B**) left knee, and (**C**) left ankle. Estimates are also compared between ErKF (IMU), cluster (Clust), and inverse kinematics (IK) methods.

**Figure A5:**
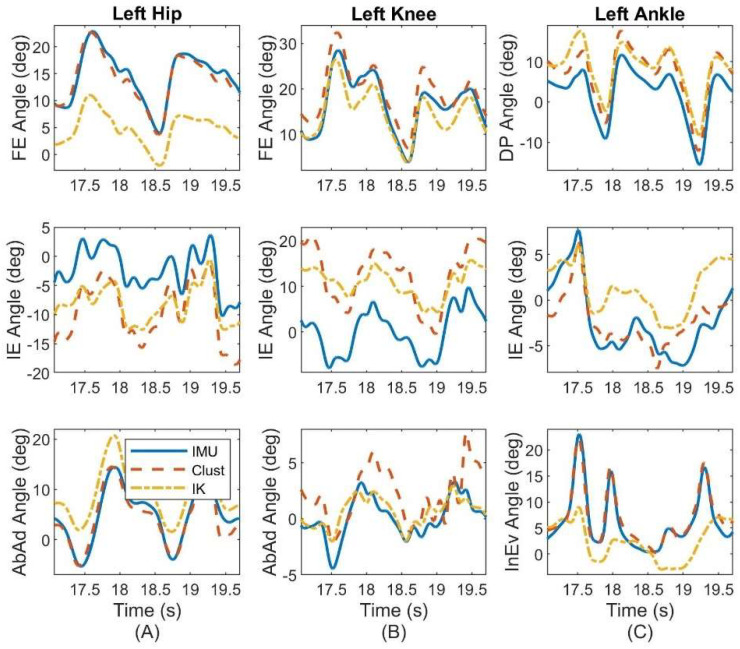
Lateral Left Walking. Example joint angle estimates over two stride cycles for lateral left treadmill walking. Plots show the three anatomical angles for (**A**) left hip, (**B**) left knee, and (**C**) left ankle. Estimates are also compared between ErKF (IMU), cluster (Clust), and inverse kinematics (IK) methods.

**Figure A6:**
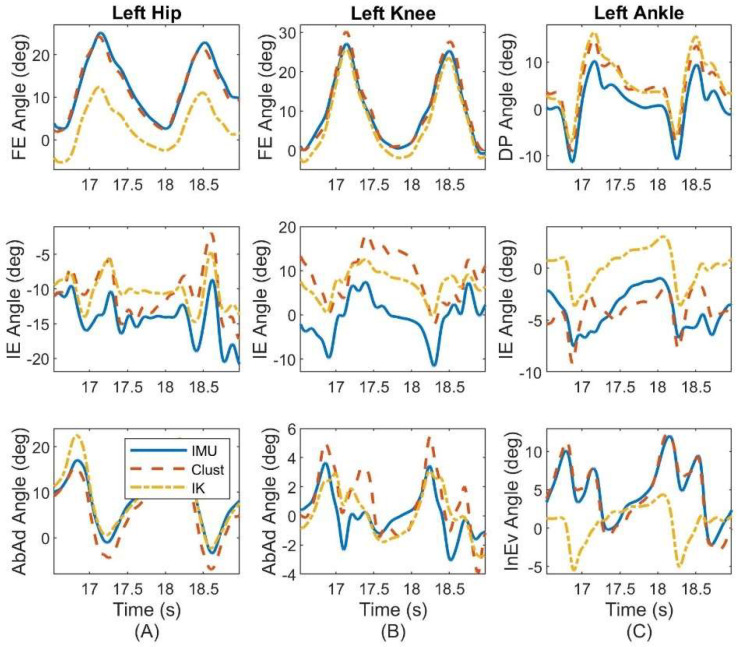
Lateral Right Walking. Example joint angle estimates over two stride cycles for lateral right treadmill walking. Plots show the three anatomical angles for (**A**) left hip, (**B**) left knee, and (**C**) left ankle. Estimates are also compared between ErKF (IMU), cluster (Clust), and inverse kinematics (IK) methods.

## Data Availability

All relevant data can be found in the Supplementary spreadsheet file, File S1.

## Supplementary Materials

The following supporting information can be downloaded at: https://www.mdpi.com/article/10.3390/s22218398/s1, File S1: Data File.
